# Synthesis, Crystal Structure, Site Occupancy, and Magnetic Properties of Aluminum-Substituted M‑Type Sr Hexaferrite SrFe_12‑x_Al_*x*_O_19_ Nanoparticles

**DOI:** 10.1021/acs.chemmater.4c02205

**Published:** 2025-01-20

**Authors:** Matilde Saura-Múzquiz, Anna Zink Eikeland, Marian Stingaciu, Henrik Lyder Andersen, Maxim Avdeev, Mogens Christensen

**Affiliations:** † Departamento de Física de Materiales, Faculty of Physics, Universidad Complutense de Madrid, Plaza de Ciencias 1, Ciudad Universitaria, Madrid 28040, Spain; ‡ Center for Materials Crystallography, Department of Chemistry and iNANO, 1006 Aarhus University, Langelandsgade 140, Aarhus C 8000, Denmark; § Instituto de Ciencia de Materiales de Madrid (ICMM), CSIC, Madrid 28049, Spain; ∥ 5419 Australian Nuclear Science and Technology Organisation (ANSTO), New Illawarra Road, Lucas Heights, NSW 2234, Australia

## Abstract

The synthesis of aluminum-substituted strontium hexaferrite nanoparticles (SrFe_12‑*x*_Al*_x_* O_19_ with *x* = 0–3), via three different preparation methods, is investigated. The synthesis methods are hydrothermal autoclave (AC) synthesis, a citrate sol–gel (SG) synthesis, and a solid-salt matrix (SSM) synthesis. Evaluation of macroscopic magnetic properties and of lattice parameters obtained by Rietveld analysis of powder X-ray diffraction (PXRD) data indicate that successful substitution of Al into the crystal structure is only achieved for the SG method. For the SG sample with *x* = 3, the coercivity was found to increase by 73% to 830 kA/m, while the saturation magnetization was reduced by 68% to 22.6 Am^2^/kg compared to the nonsubstituted *x* = 0 SG sample. The SSM and AC samples did not show any significant changes in their magnetic properties. To examine the nature of the Al insertion in detail, neutron powder diffraction (NPD) data were collected on the SSM and SG samples. Combined Rietveld refinements of the PXRD and NPD data confirm that effective substitution of the Al ions is only achieved for the SG sample and reveal that Al occupies mainly the (2*a*)_Oh_ and (12*k*)_Oh_ sites and to a lesser extent the (4*e*)_BP_, (4*f*)_Oh_ and (4*f*)_Td_ sites. Moreover, the relative degree of site occupation varies with increasing Al substitution. The intrinsic magnetization according to the refined atomic magnetic moments and Al site occupation fractions was extracted from the NPD data and compared with the measured macroscopic magnetization. A remarkable agreement exists between the two, confirming the robustness and accuracy of the Rietveld analysis.

## Graphical abstract

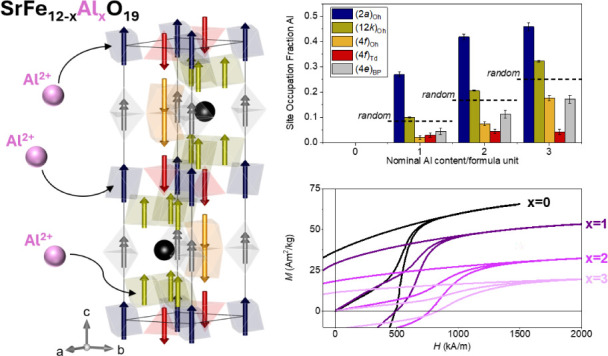

## Introduction

Rare-earth free permanent magnetic materials play a key role in many technological fields. M-type hexaferrite SrFe_12_O_19_ is a highly used rare-earth free permanent magnetic material due to its relatively low cost, high chemical stability, good magnetic properties and high Curie temperature (*T*_C_ = 737 K).^1^ The crystal structure of strontium M-type hexaferrite belongs to the centrosymmetric space group *P*6_3_/*mmc* (#194). It consists of a hexagonal close-packed arrangement of O^2^^–^ ions, following an ABAB or ACAC stacking sequence along the [001] direction. Within every five oxygen layers, one oxygen ion is substituted by a Sr^2+^ ion, causing a localized distortion in the lattice. The Fe^3+^ ions are situated in the interstitial sites between the oxygen ions, giving rise to a total of 5 iron sites; 3 of which have octahedral coordination (2*a*, 12*k*, 4*f*), one tetrahedral coordination (4*f*), and one bipyramidal coordination (4*e*). In principle, the bipyramidal site corresponds to a 2*b* Wyckoff position at the atomic coordinates x = 0, y = 0, z = 1/4. However, X-ray diffraction investigations of BaFe_12_O_19_ by Townes et al. already in 1967,^2^ as well as more recent studies,^3^ revealed that the atomic position in the bipyramidal site splits into two half-occupied atomic positions, slightly displaced from the horizontal mirror plane at z = 1/4. The Fe^3+^ at the bipyramidal site is therefore described by a half occupied 4*e* Wyckoff position, with atomic coordinates x = 0, y = 0 and z = z. The SrFe_12_O_19_ structure is illustrated in Figure 1.

**Figure 1:**
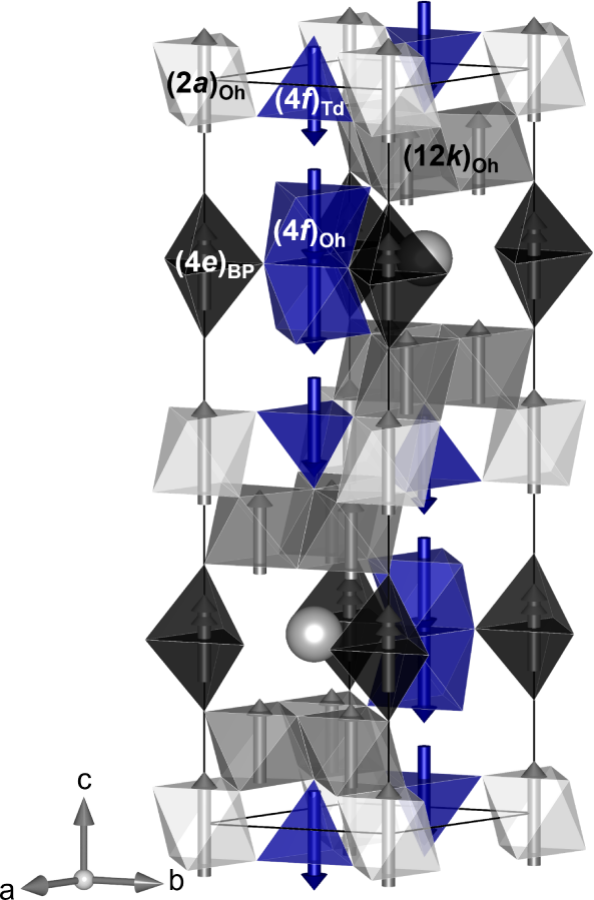
Schematic representation of one unit cell of SrFe_12_O_19_. The spin-up Fe sites are represented in gray scale polyhedra, and the spin down sites in blue polyhedral. The magnetic spins are represented by the arrows. The Sr atoms are represented by the gray spheres. The oxygen atoms (not shown) are positioned at the corners of the polyhedra. Illustration made using the software VESTA.^30^

Traditionally, SrFe_12_O_19_ is synthesized by a standard ceramic method.^4^ However, new synthesis methods are currently being investigated in order to exploit the potential of reducing the particle size to the nanoscale regime as a means to enhancing the magnetic properties of the material. Some of these synthesis methods include coprecipitation methods,^5^ sol–gel methods,^6–8^ hydrothermal methods,^9–11^ microwave induced combustion methods,^12^ and the reverse micelle technique.^13^ The produced SrFe_12_O_19_ samples show remarkably different particle shapes and sizes depending on the synthesis method used, spanning from agglomerated isotropic particles to plates, needles, or tiny superparamagnetic crystallites. The resulting magnetic properties are highly dependent on the crystallite size,^14,15^ size distribution, morphology^9,16^ and texture.^17^ To control these characteristics, a carefully designed synthesis route is essential.

A different approach to tailoring the magnetic properties of a material is by modifying its crystal structure, i.e., modifying it at the atomic level. Several studies on the partial cation substitution of M-type ferrites have previously been published, e.g., the substitution of Fe by La^3+^, Pr^3+^ or Nd^3+^ by J. F. Wang et al.,^18–20^ which gave rise to a moderate enhancement of the coercivity, *H*_c_. The most significant enhancement of coercivity to date by substitution of a single cation has been achieved by Al^3+^ substitution.^21–24^ Using an autocombustion synthesis method for preparation of SrFe_12‑*x*_Al*_x_* O_19_ followed by heat treatment, an *H*_c_ of 18.1 kOe (1440.3 kA/m) was achieved for *x* = 4 by Luo et al.^21^ A similar value of 17.75 kOe (1398.2 kA/m) was obtained by H. Z. Wang et al., also for a substitution of *x* = 4, by a glycin-nitrate method and subsequent annealing.^22^ However, the highest reported coercivity value for a ferrite material reported to date was recently published by Gorbachev et al. for Ca and Al substituted SrFe_12_O_19_, (Sr_1‑x/12_Ca_x/12_Fe_12‑x_Al_*x*_O_19_ with x = 5.5) reaching the impressive value of 36 kOe (2871 kA/m) at room temperature, and 42 kOe (3342 kA/m) at 180 K.^25–27^ These values are extraordinarily high, largely exceeding even the intrinsic coercivity values of the Nd_2_Fe_17_B magnets (∼10 kOe; 796 kA/m).^28^ However, the enhancement of coercivity diminishes the saturation magnetization, which is often reduced by 50% or more compared to nonsubstituted SrFe_12_O_19_. This can be partly explained by the inverse relation between coercivity and saturation magnetization (*H*_c_ < 2*K*_1_/μ_0_ *M*_s_), where *K*_1_ is the magnetocrystalline anisotropy constant. Given that SrFe_12_O_19_ adopts a ferrimagnetic structure, with most of the Fe^3+^ sites having spin up and two sites having spin down (illustrated in blue polyhedral in Figure 1), the reduction in magnetization could be due to aluminum occupying the up-spin (predominant spin direction) sites in the hexaferrite structure thereby reducing the overall up-spin magnetization. These were the conclusions reached by V. Dixit et al. in their first-principle total-energy calculations based on density functional theory, where they studied the site occupancy and magnetic properties of Al substituted SrFe_12_O_19_, with *x* = 0.5 and *x* = 1.^29^ The calculations indicate that the Al atoms preferentially occupy the (2*a*)_Oh_ and (12*k*)_Oh_ sites, with increasing probability of the (12*k*)_Oh_ site being occupied by Al as the synthesis temperature increases. However, until now no experimental evidence had been reported to confirm this.

In this study, the syntheses of Al-substituted Sr-ferrite (SrFe_12‑*x*_Al*_x_* O_19_, *x* = 0–3) using three different synthesis methods, namely sol–gel (SG), solid-salt-matrix (SSM) and autoclave (AC), have been investigated. The composition, crystal structure and microstructure of the obtained samples are investigated by Rietveld analysis of powder X-ray diffraction (PXRD) data. The magnetic properties of the samples are analyzed by vibrating sample magnetometry (VSM). The data indicates that successful substitution is only achieved using the SG synthesis method. To further investigate the nature and effect of the Al insertion into the structure, joint Rietveld refinements were performed on PXRD and neutron powder diffraction (NPD) data collected on both the SSM and SG samples. This data allowed us to determine the preferred substitution sites as a function of aluminum content and to understand its influence on the magnetic properties of the compound. The study advances the understanding of Al substitution in SrFe_12‑*x*_Al*_x_* O_19_ by demonstrating that significant incorporation is achieved exclusively via citrate sol–gel synthesis, in contrast to hydrothermal autoclave or solid-salt matrix methods. By systematically isolating the effects of Al substitution, our work builds upon prior studies, such as Stingaciu et al. (2021),^31^ which investigated cosubstitution of Ca, Cr, and Al in hexaferrites, and other reports that focused on Al-substituted hexaferrites but relied primarily on PXRD for structural analysis.^23,26^ By integrating high-resolution PXRD and NPD refinements, we provide unprecedented insights into the site-specific distribution of Al and its impact on the atomic magnetic structure, offering a comprehensive correlation with macroscopic magnetic properties. These findings not only resolve questions surrounding selective site occupation in Al-substituted hexaferrites but also highlight the potential of sol–gel synthesis for engineering hexaferrite materials tailored for high-performance applications.

## Experimental Methods

### Sample Preparation

Aluminum-substituted SrFe_12_O_19_ nanocrystalline samples were synthesized by three different methods: citrate sol–gel (SG), solid-salt matrix (SSM) and hydrothermal autoclave (AC) synthesis, following similar synthesis procedures to those previously reported by Eikeland et al.,^6,10^ using starting chemical reagents with purity ≥98% (Sigma-Aldrich or Chem-Solution GmbH, ACS reagent grade or higher).

### Sol–Gel Synthesis (SG)

Sol–gel synthesized SrFe_12‑*x*_Al*_x_* O_19_ was prepared using Fe­(NO_3_)_3_·9H_2_O, Sr­(NO_3_)_2_, and Al­(NO_3_)_3_·9H_2_O powders. The powders were dissolved in a small amount of distilled water under stirring. Dissolved citric acid was added to the solution after which the solution was neutralized using concentrated NH_4_OH. The ([Fe^3+^]+[Al^3+^]):[Sr^2+^] molar ratio was set to 11.5, and the nitrate to citric acid ratio was 1:1.

The solution was dried on a hot plate at 100 °C and subsequently heated to 250 °C to let the gel undergo an autocombustion reaction forming a low-density gray powder. Finally, the precursor was calcined at 925 °C for 30 min.

### Solid-Salt Matrix (SSM)

The solid-salt-matrix synthesis utilizes a solid matrix-based synthesis, where free-standing nanocrystallites grow in a NaCl matrix. The SrFe_12‑*x*_Al*_x_* O_19_ nanocrystallites were prepared by dissolving SrCl_2_·6H_2_O, FeCl_3_·6H_2_O, and Al­(NO_3_)_3_ in distilled water to obtain a ([Fe^3+^]+[Al^3+^]):[Sr^2+^] molar ratio of 11. The metal ion solution was then added to a 1 M solution of Na_2_CO_3_ under constant stirring. The synthesis was carried out with 0.5 mol of Na_2_CO_3_ per mole of Cl^–^, resulting in a 1:1 molar ratio between Na^+^ and Cl^–^. A 5.5 M solution of citric acid was finally added to the mixture. The molar ratio between citric acid and Na_2_CO_3_ was 1.5:1. The mixture was dried to a gel in a convection oven at 120 °C overnight. The dried gel was crushed in a mortar and placed in a thin layer of approximately 2 mm in a convection oven at 450 °C for 1 h to burn off the organic residues. Afterward, the precursor was calcined at 790 °C for 1 h, followed by cooling to room temperature. The product was washed in water and 4 M HNO_3_ to remove the NaCl matrix and finally dried at 80 °C.

### Autoclave Synthesis (AC)

Aqueous solutions of Sr­(NO_3_)_2_, Fe­(NO_3_)_3_·9H_2_O, and Al­(NO_3_)_3_·9H_2_O (1 M each) were mixed in the Teflon insert of a steel autoclave. Sixteen M NaOH was added slowly and dropwise to the mixture under intense stirring. A 50% excess of NaOH compared to the molar amount of NO_3_^–^ was added. The excess NaOH facilitates the formation of the red/brown metallic hydroxide precipitate. The ([Fe^3+^]+[Al^3+^]):[Sr^2+^] molar ratio was 4 and the Sr^2+^ concentration of the final precursor was 0.1 M. The use of an excess Sr/Fe ratio is based on previous studies showing that the Sr/Fe ratio influences the formation of unsubstituted Sr hexaferrite, including phase purity, crystallite size and morphology.^11,14,32^

The 175 mL Teflon lined autoclaves used as reaction chambers were inserted in a preheated convection oven at 240 °C for 4 h. The reactants were not dried in a furnace prior to the synthesis in order to remove adsorbed water and CO_2_. This led to the formation of SrCO_3_ as a subproduct. The products were washed with water and 4 M HNO_3_ to wash away SrCO_3_.

The reaction could therefore be written as

$${3Sr{\left(NO_{3}\right)}_{2}}_{\left(aq\right)}+{\left(12-x\right)Fe{\left(NO_{3}\right)}_{3}}_{\left(aq\right)}+{xAl{\left(NO_{3}\right)}_{3}}_{\left(aq\right)}+63NaOH_{\left(aq\right)}+{2CO_{2}}_{\left(g\right)}\overset{\Delta ,P}{⃗}SrFe_{12-x}{Al_{x}O_{19}}_{\left(s\right)}+{2SrCO_{3}}_{\left(s\right)}+{42NaNO_{3}}_{\left(aq\right)}+21NaOH_{\left(aq\right)}+{21H_{2}O}_{\left(aq\right)}$$

### Characterization

Powder X-ray diffraction data were collected on the as-synthesized powder samples using a Rigaku SmartLab diffractometer (Rigaku, Japan) equipped with a D/teX Ultra Si-strip detector, Cross Beam Optics (CBO) and Bragg–Brentano geometry. A Co K_α_ rotating anode source was used for the SG and SSM series, whereas the PXRD data of the AC samples were collected using a Cu K_α_ rotating anode source.

Neutron powder diffraction data were collected on the SG and SSM samples using a wavelength of 2.44 Å at ECHIDNA, the high-resolution powder diffractometer at the OPAL reactor (Australian Nuclear Science and Technology Organisation, ANSTO, Lucas Heights, New South Wales, Australia).^33^

Rietveld analysis of the diffraction data was carried out using the FullProf Suite software package.^34^ The Thompson-Cox-Hastings formulation of the pseudo-Voigt function was used to model the peak profile,^35^ and Finger’s model was used to describe the peak asymmetry due to axial divergence in terms of finite sample and detector sizes.^36^ The instrumental contribution to the peak broadening was accounted for by performing LeBail fits of a NIST LaB_6_ 660b standard^37^ measured under the same conditions as the studied samples. From these LaB_6_ fits, an instrumental resolution file was created and implemented in the refinements of the studied materials to deconvolute the instrumental and sample contributions to the peak width. For the Rietveld analysis, the zero shift, unit cell parameters, atomic positions and isotropic atomic displacement parameters were refined, as well as the Lorentzian refinable size (Y) and strain (X) parameters. The *S*_*z*_ size parameter was also refined to model the anisotropic shape of the crystallites employing the F­(*S*_*z*_) function according to the Platelet Vector Size model as implemented in FullProf.^38^ The platelet vector, **k**, was defined as (001). For Al-substituted samples, the atomic site occupation fractions were also refined. Details on the refinement of site occupation fractions of the Al-substituted samples are given in the results and discussion.

The room temperature (300 K) macroscopic magnetic properties of the samples were measured using a Quantum Design Physical Property Measurement System (PPMS) equipped with a Vibrating Sample Magnetometer (VSM). For the magnetization measurements of the powder samples, approximately 10–15 mg of sample were pressed into pellets of 3 mm in diameter using a hand-held press. The specimen was then placed in a tubular brass sample holder, tightly fit between two quartz rods, and fixed in place with Kapton tape, with the surface of the pellet perpendicular to the applied magnetic field.

## Results and Discussion

### Crystal Structure and Crystallite Size/Morphology

Figure 2 shows representative PXRD patterns and Rietveld refinements for the AC, SSM and SG samples with nominal Al-contents of *x* = 2. PXRD data and Rietveld refinements of the remaining samples can be found in Supporting Information. The Rietveld analyses confirm the formation of the hexagonal M-type hexaferrite structure (space group *P*6_3_/*mmc*, #194) in all three series (SG, SSM and AC) and across the entire substitution range. Notably, the AC samples with *x* = 0 and *x* = 0.5 nominal Al-contents were found to contain minor quantities of hematite impurity (α-Fe_2_O_3_), amounting to 1.9(1) wt % and 2.2(1) wt %, respectively. This impurity was also present in some of the SG samples in very small amounts (1.3(2) wt % in *x* = 1, 1.8(2) wt % in *x* = 2 and 1.93(2) wt % in *x* = 3). These minor impurity levels are negligible in influencing the observed properties, as the samples are primarily composed of the hexaferrite phase. The impurities are only detectable due to the high quality of the PXRD data and the rigorous Rietveld analysis employed in this study.

**Figure 2:**
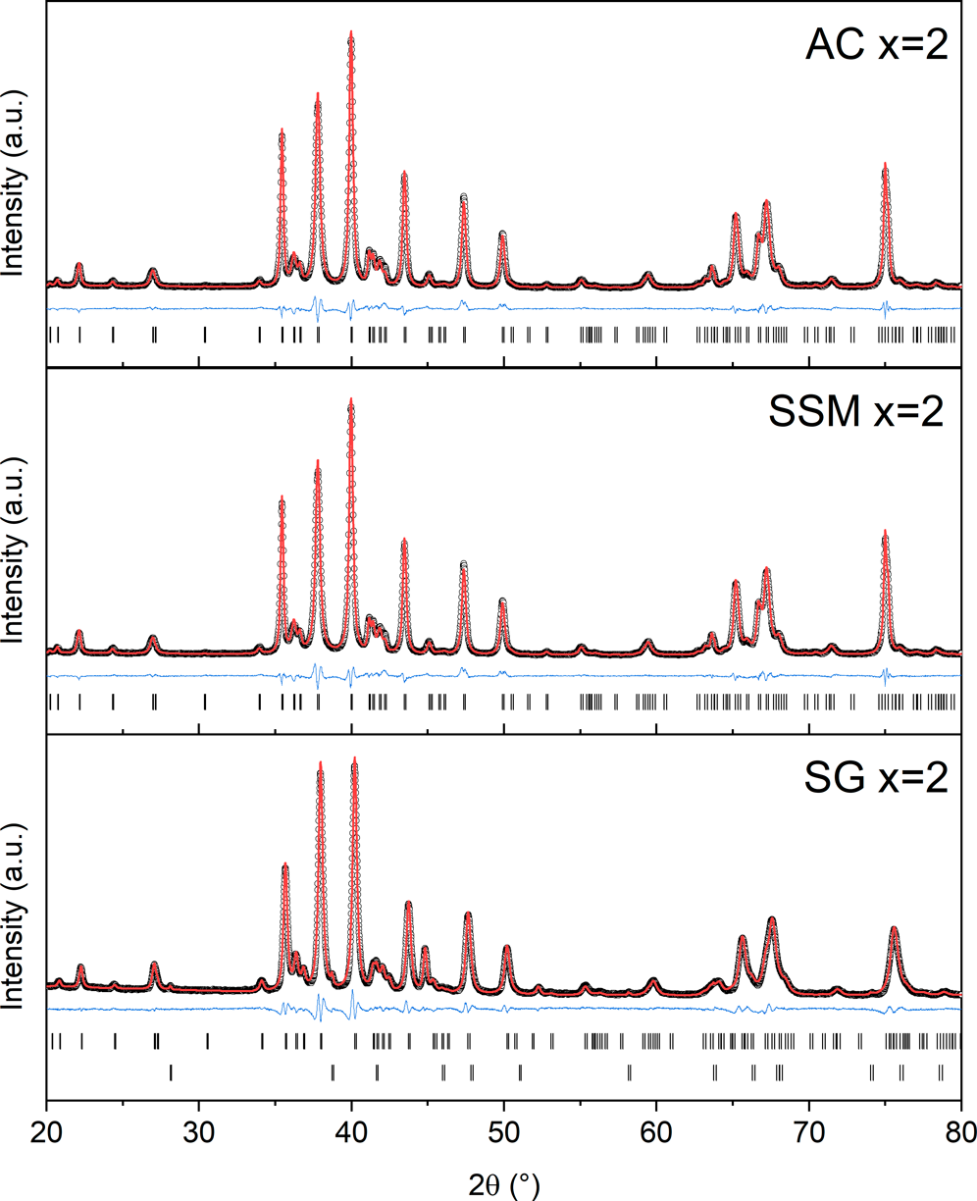
PXRD data (black dots) and refinement model (red line) of SrFe_12‑*x*_Al*_x_* O_19_ samples with nominal *x* = 2 synthesized by AC, SSM and SG. The difference between the observed and calculated intensities is given in blue, and the black bars mark the position of the Bragg reflections. Note that when NPD data was available (SSM and SG samples), a combined refinement of the structural model to the PXRD and NPD was carried out, although only the PXRD data is shown here.

As demonstrated in previously reported studies,^6,10,17^ the undoped SrFe_12_O_19_ samples exhibit differences in crystallite size and platelet aspect ratio depending on the synthesis method and reaction conditions employed. In the present study, the crystallite size and morphology have been extracted from Rietveld refinement of powder diffraction data using the platelet-vector-size model, as described in the experimental section. The hydrothermal AC method is found to yield relatively thin platelet-shaped crystallites with a thickness, *D*_c_, of 41.0(2) nm and diameter, *D*_a_, of 154(2) nm. In contrast, the SrFe_12_O_19_ crystallites prepared by the SG and SSM methods exhibit a more isotropic morphology with diameters of 73.5(5) and 60.0(2) nm and thicknesses of 63(1) and 23.76(9) nm, respectively.

Figure 3 shows the refined crystallite sizes along the *a-* and *c-*axes respectively as a function of nominal Al-content for the three series (see Table 1 for numeric values). In the AC series, no clear trend in crystallite size is observed with increasing Al content, although an overall decrease in size along the *a*-axis (*D*_a_) can be seen from 154(2) nm at x = 0 to 106.4(9) nm at *x* = 3. However, these sizes are very close to the resolution limit of the diffractometer, so the obtained sizes and trends in the *D*_a_ of AC samples should not be overinterpreted. In the SSM series, no significant change in crystallite size is observed with increasing nominal Al-content along any of the axes of the platelets (*D*_a_ ∼ 50–60 nm, *D*_c_ ∼ 22–24 nm). In the case of the SG samples, an initial decrease in crystallite size is observed for the *x* = 1 sample (*D*_a_ = 61(1) nm, *D*_c_ = 51(1) nm). However, higher levels of Al-content lead to an increase in crystallite size along both axes that is particularly pronounced along the *c*-axis (for *x* = 3, *D*_a_ = 86(6) nm, *D*_c_ = 85 (8) nm), as illustrated in Figure 3.

**Figure 3:**
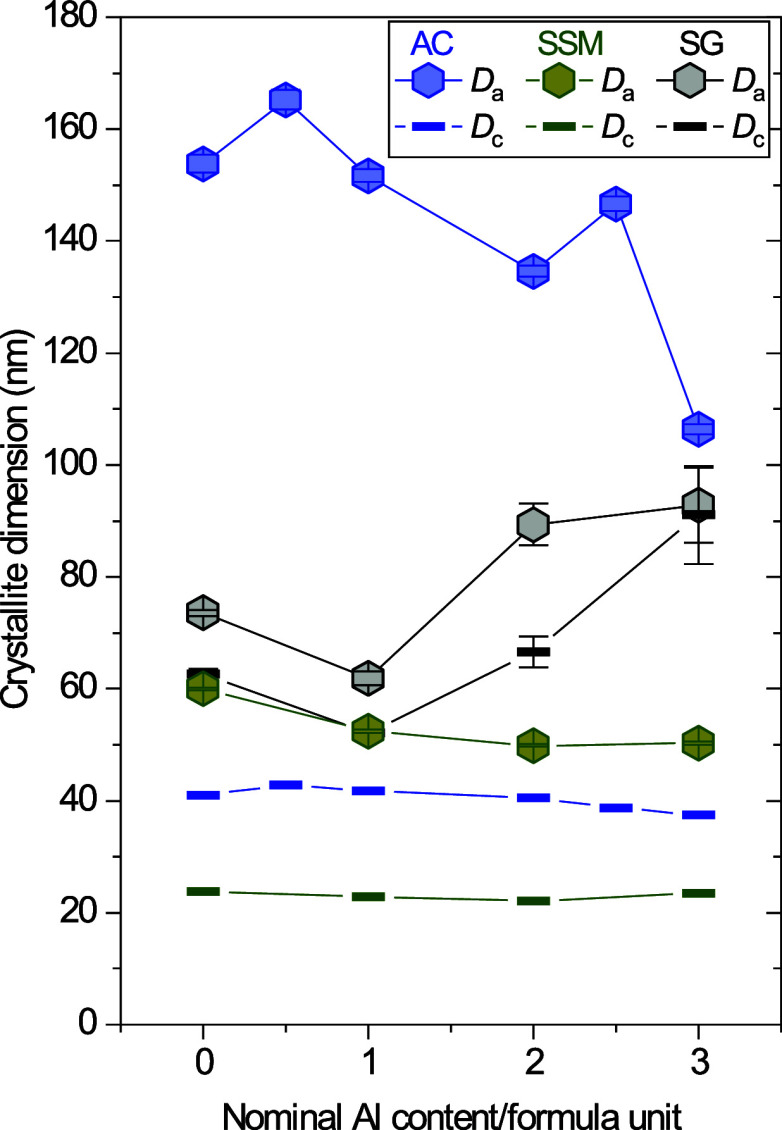
Variation of crystallite sizes along the crystallographic *a*-axis (*D*_a_) and the crystallographic *c*-axis (*D*_c_) with increasing nominal aluminum content, for samples synthesized by the three different methods, AC (blue), SSM (green) and SG (gray). Where not shown, the errors are smaller than the size of the symbols.

**Table 1:** Refined Lattice Parameters *a -Axis* (Å) and *c-Axis* (Å), Crystallite Sizes along the *a*-Axis, *D*_a_ (nm), and *c*-Axis, *D*_c_ (nm), as well as Saturation Magnetization, *M*_s_ (Am^2^/kg), Remanent Magnetization, *M*_r_ (Am^2^/kg), Squareness Ratio (*M*_r_/*M*_s_) and Coercive Field *H*_c_ (kA/m) for All Samples, with x Being the Nominal Al Content/Formula Unit (SrFe_12‑*x*_Al*_x_* O_19_)­^a^

*x*	*a-axis* (Å)	*c-axis* (Å)	*D*_a_ (nm)	*D*_c_ (nm)	*D*_a_/*D*_c_	*M*_s_ (Am^2^/kg)	*M*_r_ (Am^2^/kg)	*M*_r_ */M*_s_	*H*_c_ (kA/m)
AC									
0	5.88245(2)	23.0743(1)	154(2)	41.0(2)	3.75(5)	64.4(2)	41.5(1)	0.644(2)	164(3)
0.5	5.88212(2)	23.0723(1)	165(2)	42.8(2)	3.86(5)	64.5(1)	43.43(9)	0.674(2)	173(2)
1	5.88349(2)	23.0762(1)	152(1)	41.7(2)	3.64(3)	67.0(1)	50.23(9)	0.750(2)	143(2)
2	5.88166(2)	23.0684(1)	134(1)	40.5(2)	3.32(3)	63.1(1)	44.07(8)	0.698(2)	162(2)
2.5	5.88338(2)	23.080(1)	147(1)	38.7(2)	3.79(4)	63.4(4)	47.9(1)	0.754(5)	125(1)
3	5.88186(2)	23.0728(1)	106.4(9)	37.4(2)	2.84(3)	63.3(1)	44.13(9)	0.697(2)	144(2)
SSM									
0	5.88637(2)	23.0590(1)	60.0(2)	23.76(9)	2.53(1)	70.4(2)	38.83(7)	0.551(2)	482(3)
1	5.88062(4)	23.0366(3)	52.4(3)	22.9(2)	2.29(2)	61.9(2)	34.51(6)	0.557(2)	549(13)
2	5.87847(5)	23.0268(3)	49.9(3)	22.1(2)	2.26(2)	62.1(2)	34.48(5)	0.555(2)	568(5)
3	5.88076(5)	23.0351(3)	50.3(3)	23.5(2)	2.14(2)	66.0(2)	36.54(6)	0.554(2)	519(3)
SG									
0	5.88187(3)	23.0654(2)	73.5(5)	63(1)	1.17(2)	71.6(2)	36.56(4)	0.511(2)	479(6)
1	5.86027(5)	22.9901(3)	61(1)	51(1)	1.18(4)	57(1)	29.13(6)	0.51(1)	492(13)
2	5.84179(8)	22.9317(4)	86(3)	65(3)	1.34(8)	36.6(3)	18.37(2)	0.502(5)	765(18)
3	5.8190(1)	22.8574(6)	86(6)	85(8)	1.0(1)	22.6(3)	11.442(8)	0.506(6)	830(21)

a Values of *M*_s_ (Am^2^/kg), *M*_r_ (Am^2^/kg), *M*_r_/*M*_s_ and *H*_c_ (kA/m) obtained at 300 K from VSM measurements.

### Evidence for Al-Substitution—Lattice Parameters and Magnetic Properties

Successful isostructural substitution of Al for Fe in the hexaferrite structure would be expected to cause a Vegard’s law-like behavior with a gradual reduction in the lattice parameters as a function of increasing Al content, due to the smaller effective ionic radius of Al^3+^ compared to Fe^3+^ in tetrahedral (0.39 vs 0.49 Å, ∼ 20% smaller), octahedral (0.535 vs 0.645 Å, ∼ 18% smaller) and bipyramidal (0.48 vs 0.58 Å, ∼ 20% smaller) coordination.^39^ The unit cell axes of the Al-doped SrFe_12_O_19_ samples (SrFe_12‑*x*_Al*_x_* O_19_) of the three series (AC, SSM and SG) extracted from Rietveld refinements of PXRD and NPD data, are shown in Figure 4, and the numerical values are given in Table 1. In the case of the AC series, no notable changes in the unit cell axes are observed with increasing Al content. This lack of change suggests that no (or only negligible) effective Al substitution is taking place in those samples. In the case of the SSM series, a slight decrease in unit cell axes is observed with increasing Al substitution, with the smallest unit cell axes found at compositions of *x* = 2. This small change in unit cell parameters could indicate that some Al insertion is taking place in these samples, although if so, it is very minor. The SG samples, however, exhibit a clear linear decrease of the unit cell axes with increasing Al content, following Vegard’s law both along the *a*- and *c*-axes.^40^ These results suggest that, unlike the AC and SSM samples, Al is effectively substituted into the SrFe_12_O_19_ structure of the SG-synthesized samples.

**Figure 4:**
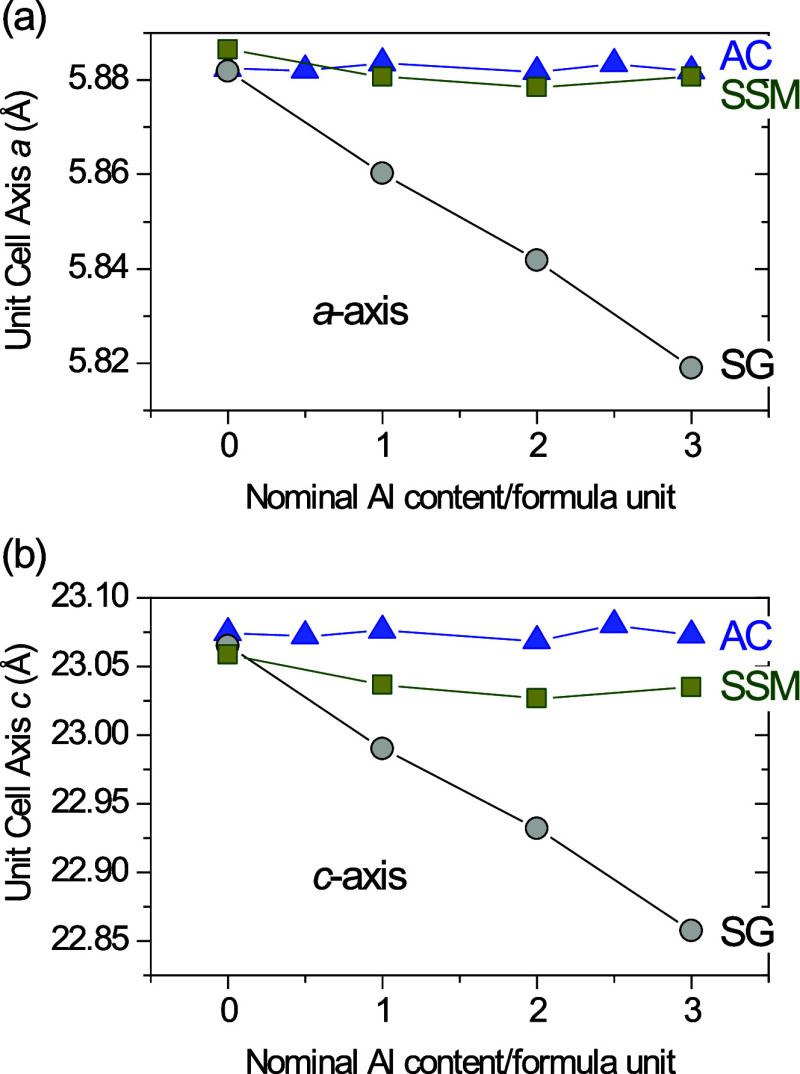
Unit cell (a) *a*-axis and (b) *c*-axis as a function of nominal Al-content, extracted from Rietveld analysis of NPD and PXRD data, for samples synthesized by the three different methods; AC (blue), SSM (green) and SG (gray). When not shown, the errors are smaller than the size of the symbols.

### Magnetic Properties

These observations from the refined unit cell parameters are corroborated by the macroscopic magnetic behavior of the samples as illustrated by the field-dependent magnetization curves (mass magnetization, *M*, vs effective field, *H*_eff_) shown in Figure 5. Successful substitution of nonmagnetic Al^3+^ ions into the structure would be expected to cause a considerable change in the degree of magnetization that can be induced in the samples. The magnetization would be expected to decrease or increase depending on whether Al occupies crystallographic up-spin sites (predominant spin direction) or down-spin sites, respectively (see Figure 1). Here, little-to-no change in the magnetic behavior is observed for both the AC and SSM samples with increased nominal Al content, confirming the lack of (or negligible degree of) Al^3+^ insertion into the structure. In contrast, the SG samples exhibit a clear and gradual change in magnetic behavior with lower magnetization and higher coercivity as the nominal content of Al increases.

**Figure 5:**
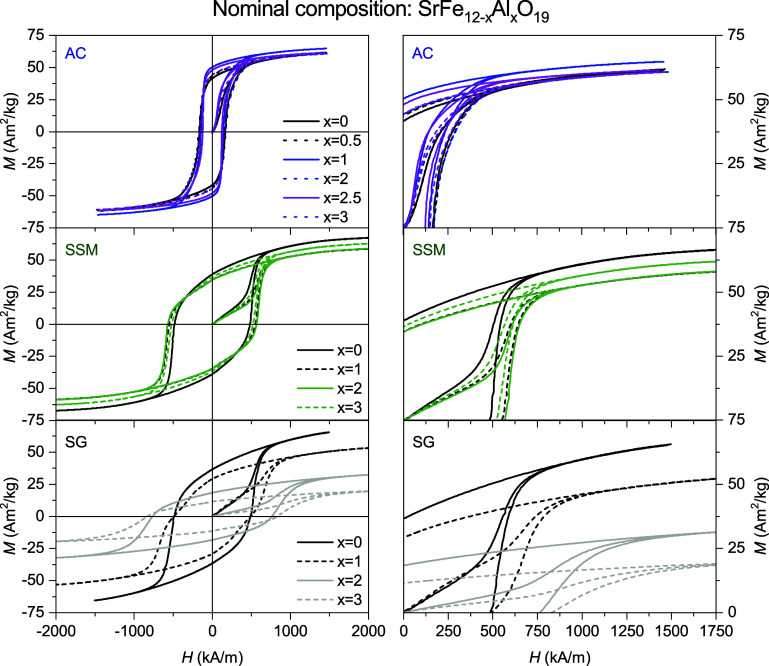
Room temperature (300 K) hysteresis curves (Mass magnetization, *M*, vs effective field, *H*_eff_) of the samples from the three different synthesis series (SG, SSM and AC) with different degrees of nominal aluminum substitution. Right hand-side: magnification of the fist quadrant of hysteresis curves.

The extracted values for coercivity (*H*_c_), saturation magnetization (*M*_s_), and remanent magnetization (*M*_r_) are shown in Figure 6 and given in Table 1. It should be noted that the saturation magnetization value reported here is not the measured magnetization value at the maximum applied field. Rather, *M*_s_ is extracted using the law of approach to saturation,^41^ where the measured magnetization of the sample is extrapolated to calculate the true saturation value of the sample. This obtained *M*_s_ is sometimes referred to here as “*measured M*_s_” to distinguish it from the “*calculated M*_s_” using the refined magnetic moments from NPD data (discussed in the next section).

**Figure 6:**
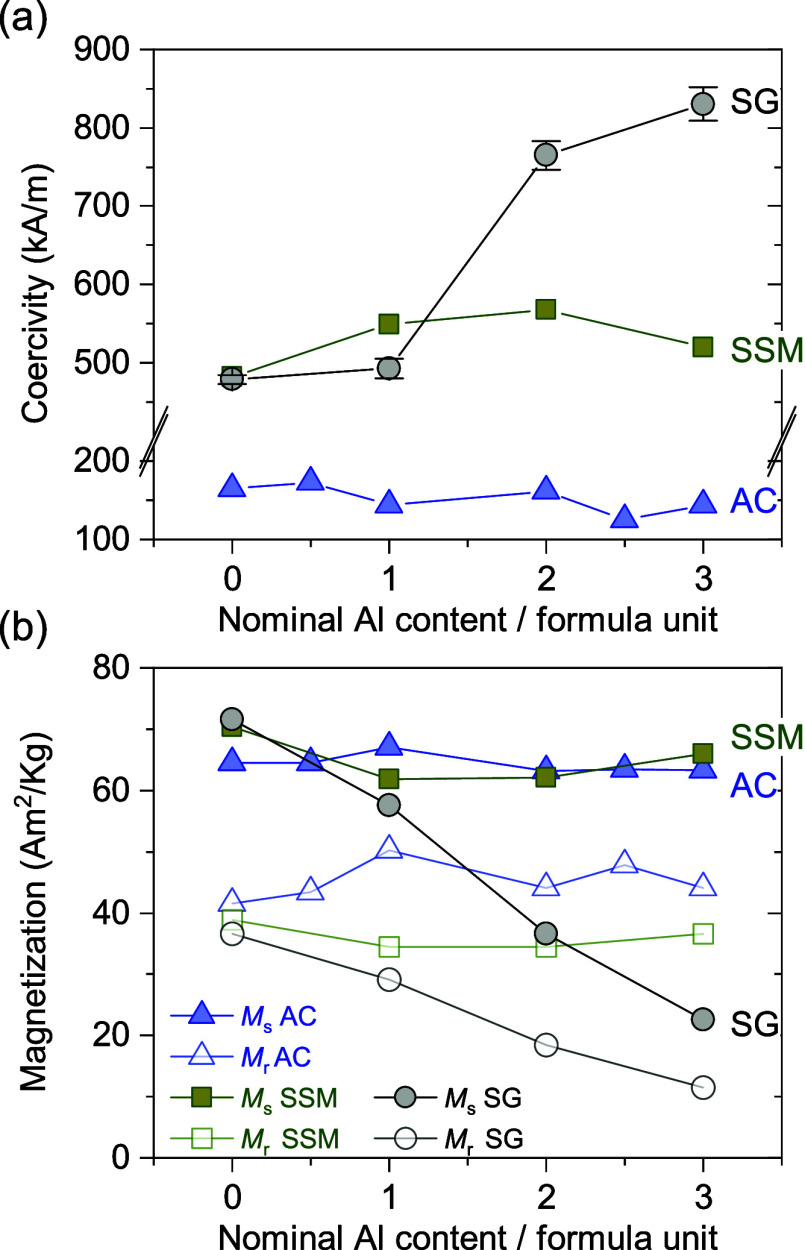
(a) Coercivity and (b) saturation and remanent magnetization of all samples extracted from the measured hysteresis curves, plotted as a function of nominal aluminum content per formula unit. When not shown, the errors are smaller than the size of the symbols.

In the SG series, where effective insertion of the Al^3+^ cation takes place, an enhancement of the coercivity is observed as the Al content increases, from 479(6) kA/m at *x* = 0 to 830(21) kA/m at *x* = 3. The coercivity enhancement is accompanied by a decrease of the saturation magnetization, in accordance with previously reported studies and theoretical calculations,^29^ from 71.6(2) Am^2^/kg at *x* = 0 to 22.6(3) Am^2^/kg at *x* = 3.

A somewhat similar trend in coercivity and saturation magnetization is observed for the SSM series from *x* = 0 to *x* = 2. The changes in magnetic properties with increasing Al content are, however, much less pronounced than that of the SG samples. Nevertheless, these results could indicate that a very small amount of Al is present in the hexaferrite structure of the SSM samples, although it seems to reach a maximum degree of substitution at *x* = 2, after which the coercivity decreases and the saturation increases, reaching values closer to that of the undoped sample. These results are consistent with the refined unit cell parameters of the SSM samples, where a small decrease in both the *a-* and *c-*axes were observed, attaining the smallest unit cell at *x* = 2. For the AC samples, no considerable change in the magnetic properties is observed with increasing Al-content, confirming once again the lack of successful substitution of Al into the structure.

As mentioned earlier, small amounts of α-Fe_2_O_3_ were detected in some AC samples (x = 0 and x = 0.5) and SG samples (x = 1, 2, and 3). The presence of α-Fe_2_O_3_ as a secondary phase could affect the magnetic properties of the samples. α-Fe_2_O_3_, being antiferromagnetic, can weaken the overall magnetic response by reducing coercivity and saturation magnetization through weak magnetic interactions with the hexaferrite phase. This could result in diluted magnetization and diminished magnetic performance. However, in all cases, the α-Fe_2_O_3_ content is very low (<3 wt %), and its impact on the overall magnetic properties is negligible. No significant differences in magnetic properties are observed between the AC samples where hematite is formed (x = 0 and x = 0.5), and the rest of the AC samples, likely due to the minimal α-Fe_2_O_3_ content. In the SG samples, large variations in saturation magnetization are observed but as discussed above, these are primarily attributed to the increasing Al content, which reduces the net magnetic moment within the SrFe_12_O_19_ structure. Any minor effects from the small amount of α-Fe_2_O_3_ impurities (<2 wt %) are not discernible against the dominant influence of Al substitution.

As previously mentioned, parameters such as particle size, morphology, and texture have a significant influence on the magnetic properties of materials. This can also be observed here. In addition to the trends in magnetic properties within each series, the data in Table 1 show a clear correlation between the aspect ratio of the nanoparticles (*D*_a_/*D*_c_) and the attained *M*_r_/*M*_s_. The highest *M*_r_/*M*_s_ ratio (0.70) is reached for the AC series, where the more anisotropic particles are obtained, with an average *D*_a_/*D*_c_ of 3.53. This is followed by a *M*_r_/*M*_s_ of 0.55 for the SSM series, of average *D*_a_/*D*_c_ of 2.30, and the smallest *M*_r_/*M*_s_ of 0.51 is observed in the SG series, of average *D*_a_/*D*_c_ equal to 1.18. This result is consistent with our previous studies of hexaferrite nanoparticles, and the influence of morphology on magnetic texture.^9,17^ Ideally, to isolate the contribution of Al substitution on the magnetic properties from other effects, it would be necessary to compare samples with identical Al content but varying structural features (e.g., crystallite size, morphology, size distribution). This was the aim of the present study, where we attempted to achieve comparable levels of Al incorporation in nanoparticles synthesized through different methods. However, the lack of effective Al substitution in the SrFe_12_O_19_ structure in the AC and SSM series prevented this.

Alternatively, the isolated effect of Al substitution could be analyzed by comparing particles with varying degrees of Al content but identical structural characteristics. The SG series, where successful Al incorporation was achieved, presented an opportunity to investigate this effect. Nonetheless, a degree of variation in crystallite dimensions is also observed across the SG series with increasing Al content. The influence of structural differences on magnetic properties is evident when comparing the nonsubstituted samples (x = 0) from the three series. For instance, the AC series exhibits distinct hysteresis curve shapes and magnetic properties compared to the SSM and SG series (Figure 5, Table 1). However, no significant differences in magnetic properties are observed between the SSM and SG x = 0 samples, despite slight variations in crystallite sizes, aspect ratios, and unit cell parameters (Figure 3, Table 1). These observations suggest that the modest variations in structural features within the SG series may have a smaller impact on the magnetic properties compared to the effects of Al substitution.

### Aluminum Site Occupancy and Magnetic Structure—Combined NPD and PXRD Rietveld Refinements

In order to examine the nature of the Al insertion and its influence on the magnetic structure, NPD data were collected for the SG and SSM series. A representative combined Rietveld refinement of the NPD and PXRD data of the SG sample for *x* = 2 (i.e., SrFe_10_Al_2_O_19_) is shown in Figure 7.

**Figure 7:**
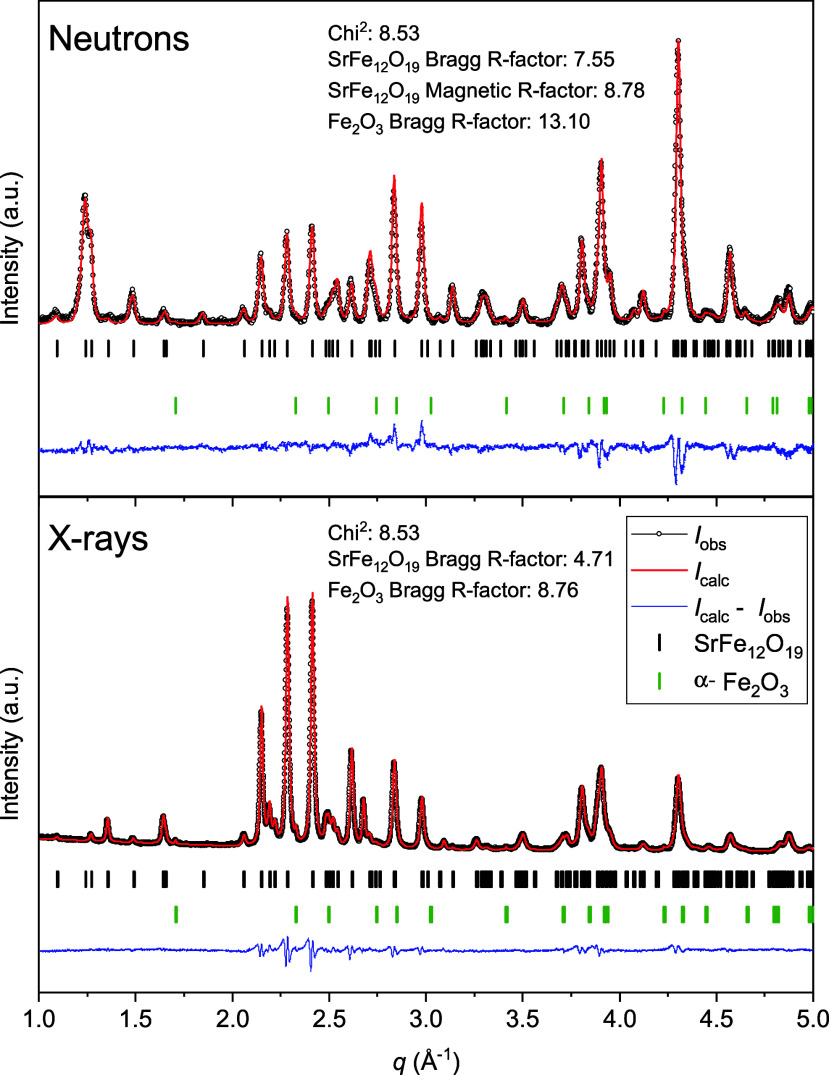
NPD (top) and PXRD (bottom) patterns, and combined Rietveld refinement of SG-synthesized sample with nominal Al substitution of *x* = 2 (i.e., SrFe_10_Al_2_O_19_).

To determine which crystallographic sites are occupied by Al in the SG SrFe_12‑*x*_Al*_x_* O_19_ samples, the following refinement strategy was used: Each of the Fe Wyckoff sites were allowed to be populated by either Fe or Al. Linear restraints were imposed on all the Fe/Al sites to avoid unphysical over or under population of each of the sites, while keeping the overall nominal stoichiometric ratio between Fe and Al within the structure fixed (i.e., 12:0, 11:1, 10:2 and 9:3 for *x* = 0, 1, 2, and 3, respectively). The total amount of aluminum was initially distributed evenly on each of the Fe sites. The relative occupation fraction of each site was then refined, allowing the overall amount of Al to be distributed freely among the different sites. The refined site occupation fractions of Al in each of the crystallographic Wyckoff sites are shown in Figure 8. The remaining occupation fraction is occupied by Fe. The numeric values, atomic coordinates, isotropic displacement parameter and refined magnetic moment of SG sample with *x* = 2 are given in Table 2. Equivalent tables of the remaining samples can be found in the Supporting Information. The refinements clearly indicate that Al preferentially occupies the octahedral 2*a* site, (2*a*)_Oh_. This site preference is followed by the (12*k*)_Oh_ site. These two sites are significantly more occupied by Al than the remaining three, and both have the atomic magnetic dipolar moment along the predominant direction (here considered as magnetic moment pointing up).

**Figure 8:**
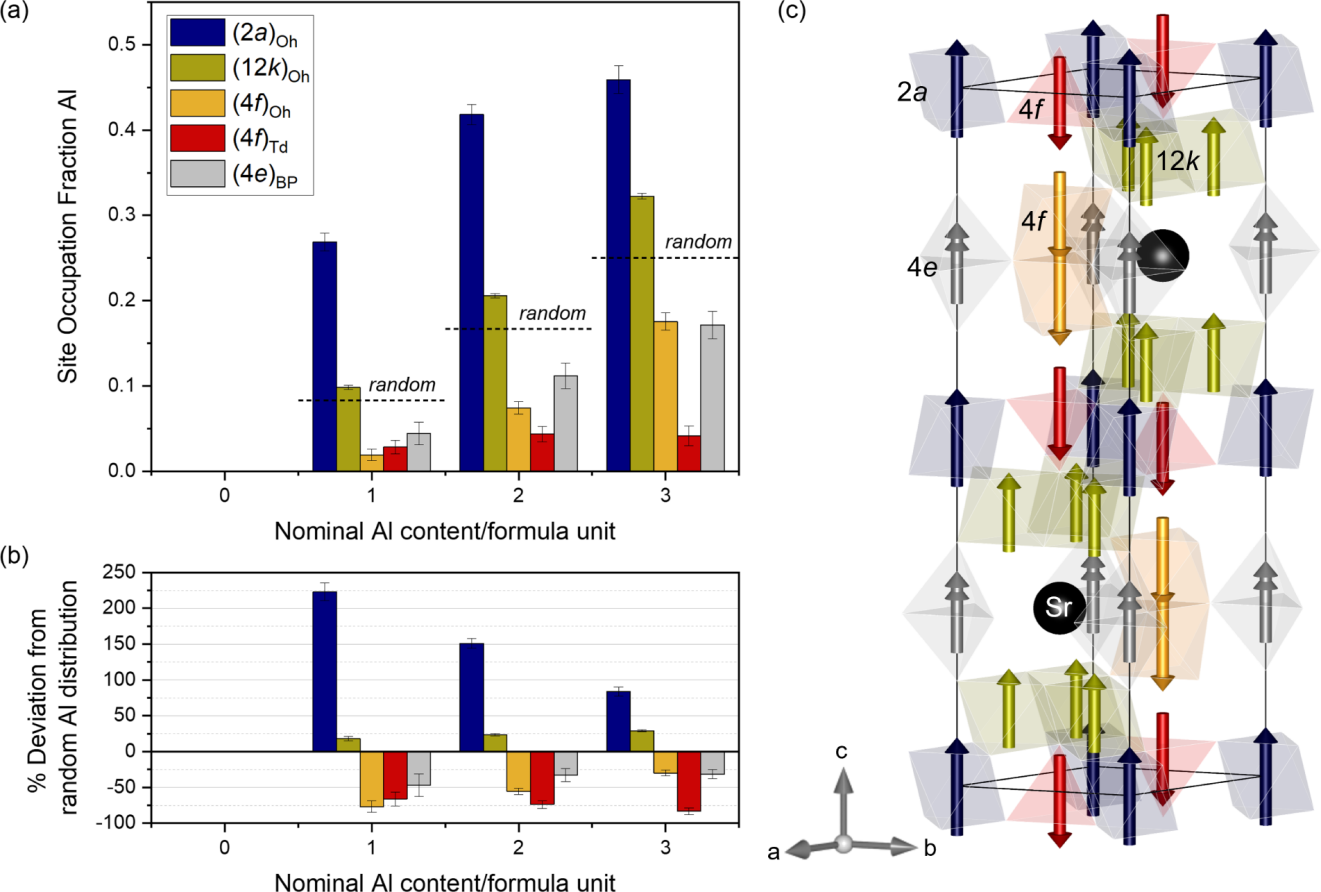
a) Refined site occupation fraction of Al cations in each of the 5 crystallographic sites for the SG-synthesized samples. b) Percentage deviation from random distribution of Al cations in the 5 sites. c) Illustration of the crystal and magnetic structure of SrFe_12_O_19_ with the crystallographic sites color-coded in accordance with a) and b). The two gray arrows in the bipyramidal site (shown in gray) correspond to the moment of the two half occupied atoms on the 4*e* site. Equivalent figures to a) and b) for the SSM samples can be found in Figure S11.

**Table 2:** Refined Crystallographic Parameters for Aluminum-Substituted SrFe_12‑*x*_Al*_x_* O_19_ (*x* = 2) Synthesized by the SG Method^a^

SG – SrFe_12‑*x*_Al*_x_* O_19_ (*x* = 2)			Refined composition:		SrFe_10.00(4)_Al_2.00(4)_O_19_				
Space group	*P*6_3_/*mmc* (No. 194)								
a = b	5.84179(8) Å								
c	22.9317(4) Å								
α=β	90°								
γ	120°								
Y	0.100(4)								
S_*z*_	0.38(4)								
X	0.33(1)								

**Atom and site**	**x**	**y**	**z**	*B*_**iso**_ **(Å** ^**2**^ **)**	**Occ.**	**Mult.**	**Compos.**	**Fraction**	*R*_*z*_ **(μ**_**B**_ **)**
Sr1	0.3333	0.6667	0.75	1.83(9)	0.08333	2	2	1	
Fe1 (2*a*)_Oh_	0	0	0	1.81(4)	0.048(1)	2	1.15(3)	0.57(1)	5.3(5)
Al1 (2*a*)_Oh_	0	0	0	1.81(4)	0.035(1)	2	0.85(3)	0.43(1)	
Fe2 (12*k*)_Oh_	0.1683(5)	0.337(1)	–0.10844(7)	1.81(4)	0.397(1)	12	9.54(3)	0.795(3)	3.0(2)
Al2 (12*k*)_Oh_	0.1683(5)	0.337(1)	–0.10844(7)	1.81(4)	0.103(1)	12	2.46(3)	0.205(3)	
Fe3 (4*f*)_Oh_	0.33333	0.66667	0.1895(2)	1.81(4)	0.154(1)	4	3.69(3)	0.923(8)	–3.9(2)
Al3 (4*f*)_Oh_	0.33333	0.66667	0.1895(2)	1.81(4)	0.013(1)	4	0.31(3)	0.077(8)	
Fe4 (4*f*)_Td_	0.33333	0.66667	0.0268(2)	1.81(4)	0.162(2)	4	3.89(4)	0.973(10)	–3.7(2)
Al4 (4*f*)_Td_	0.33333	0.66667	0.0268(2)	1.81(4)	0.004(2)	4	0.11(4)	0.027(10)	
Fe5 (4*e*)_BP_	0	0	0.249(6)	1.81(4)	0.072(1)	4	1.73(3)	0.432(8)	2.3(3)
Al5 (4*e*)_BP_	0	0	0.249(6)	1.81(4)	0.011(1)	4	0.27(3)	0.068(8)	
O1 (*4e*)	0	0	0.1500(4)	0.67(5)	0.16667	4	4	1	
O2 (*4f*)	0.33333	0.66667	–0.0552(4)	0.67(5)	0.16667	4	4	1	
O3 (*6h*)	0.184(2)	0.368(3)	0.25	0.67(5)	0.25	6	6	1	
O4 (*12k*)	0.158(1)	0.316(2)	0.0518(2)	0.67(5)	0.5	12	12	1	
O5 (*12k*)	0.506(1)	0.012(3)	0.1506(2)	0.67(5)	0.5	12	12	1	

a The Sr and O atoms were refined with full occupancy. The positive/negative sign in the refined magnetic moment indicates the direction of the magnetic moment along the z-axis in that site. Equivalent tables for the remaining samples are provided in Supporting Information.

For the SSM samples, a slightly different refinement strategy was followed. Given the very subtle variation of unit cell parameters and magnetic properties in the SSM series, the assumption of nominal Al insertion in the structure was deemed unfeasible. Thus, to obtain an estimated amount of Al insertion, the relative change in unit cell parameters of the SSM samples with respect to the unsubstituted SSM sample was used. For this, the relative change in both, *a-* and *c-*axes was calculated, and the average value of both was compared to that of the SG series, assuming that the obtained relative change in the SG series lattice parameters corresponds to the expected change for the nominal Al insertion (i.e., *x* = 1, *x* = 2 and *x* = 3), and that an equal relative change in unit cell axes would take place for the SSM and SG series. The amount of Al, i.e., *x* in SrFe_12‑*x*_Al*_x_* O_19_, was then estimated to be approximately 0.25, 0.40, and 0.30 for the Al-substituted SSM samples with nominal *x* = 1, 2 and 3, respectively. Once *x* was estimated, a similar refinement strategy to that of the SG series was followed. The corresponding amount of Al was evenly distributed among the five Fe Wyckoff sites, which were allowed to be populated by either Fe or Al. The refinements initially gave rise to negative or negligible Al occupation in all Fe sites except for the octahedral 2*a* and 12*k* sites (represented in blue and green in Figure 8b). Notably, these are the same sites found to be preferred by Al in the SG-synthesized samples (see Figure 8). Therefore, for the final refinements of the SSM series, the Al content was fixed to zero on all sites except the octahedral 2*a* and 12*k* sites. The refined *x* value of 0.25(1), 0.40(1) and 0.30(1), translates into a (10 ± 1)%, (13 ± 1)% and (11 ± 1)% occupation of Al on the 2*a* site and a (2.5 ± 0.1)%, (4.4 ± 0.2)% and (3.2 ± 0.1)% on the 12*k* site for the samples with nominal *x* = 1, 2 and 3, respectively (see tables and figures in Supporting Information). It should be noted that this amount of Al is very small, and similar quality refinements could be obtained considering no Al present in the samples.

Considering the above, the unit cell parameters, crystallite sizes and magnetic properties of all samples may be reevaluated as a function of refined Al content rather than nominal Al content. The results are shown in Figure 9. As explained in the refinement strategy, the refined Al content per formula unit of the SG samples corresponds to the nominal content (i.e., 0, 1, 2, 3), while it corresponds to zero for all samples in the AC series, and to 0, 0.25, 0.40, and 0.30 in the SSM series.

**Figure 9:**
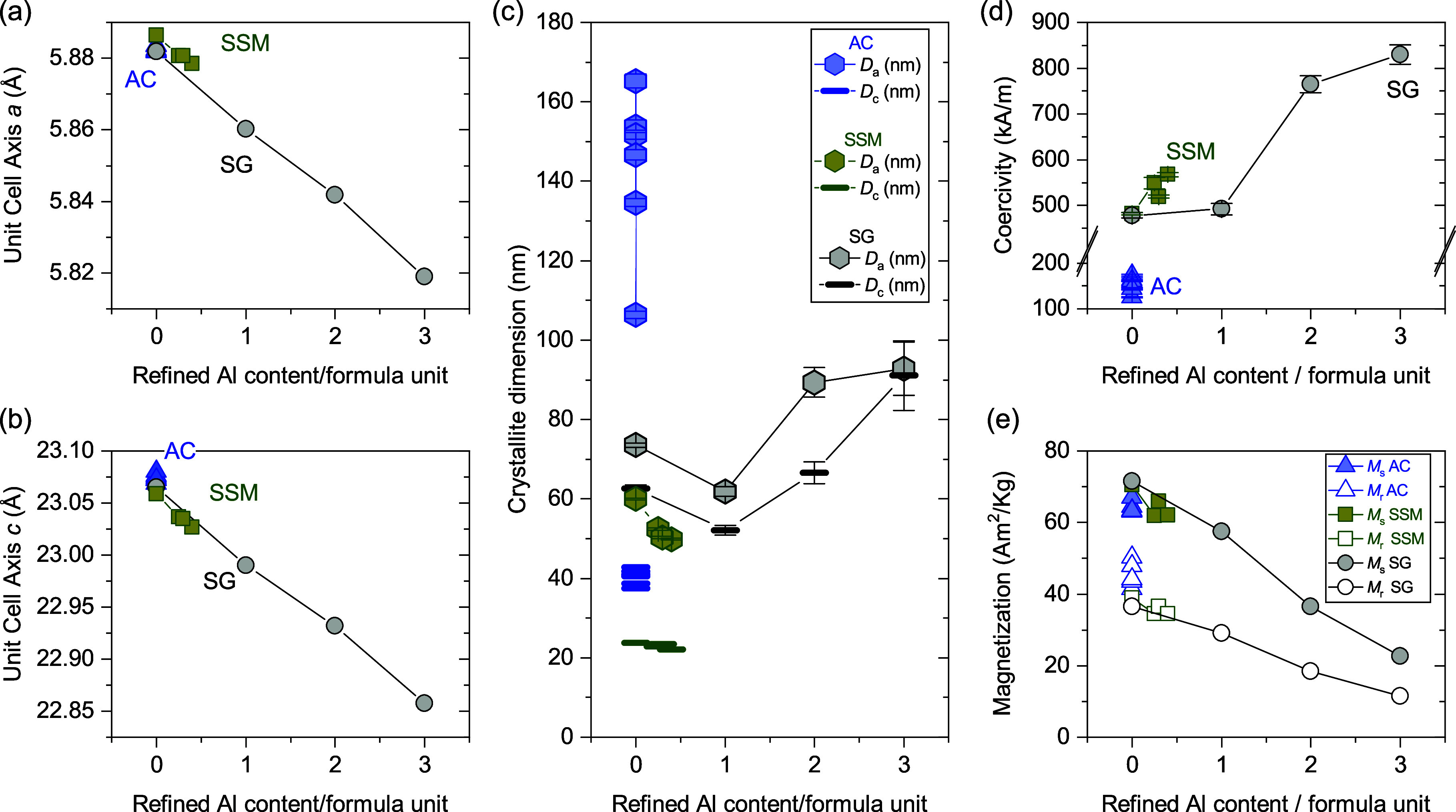
Refined (a) unit cell *a*-axis, (b) unit cell *c*-axis and (d) crystallite size of all samples as a function of refined Al content per formula unit. (d) Coercivity and (e) magnetization of all samples, plotted as a function of refined Al content per formula unit. Where no error bars are shown, the errors lie within the size of the symbols.

Figure 9 highlights that the unit cell parameters of the AC samples remain consistent across the series, as already shown in Figure 4. However, the refined crystallite sizes along the *a*-axis (*D*a) for the AC samples exhibit the largest dispersion among all series. Given that these sizes are close to or above the resolution limit of the technique, small variations in peak widths can result in significant differences in the refined sizes. Therefore, the observed dispersion in *D*a values may represent the true deviation in the measurements. Interestingly, in the case of the SSM series, the trends observed in all parameters, i.e., unit cell, crystallite sizes and magnetic properties, align with that of the SG series within the low substitution range. These results support the idea that a certain degree of Al substitution, albeit very low, is indeed taking place in the SSM series.

The results are in good agreement with the theoretical calculations by V. Dixit et al.,^29^ which predict the (2*a*)_Oh_ and (12*k*)_Oh_ to be the preferred substitution sites for Al. However, according to their calculations, which are restricted to an Al content of x = 0.5 and x = 1, the preference for Al occupying the (2*a*)_Oh_ or (12*k*)_Oh_ site varies with annealing temperature during synthesis. For the synthesis temperature employed here in the SG and SSM samples (925 and 790 °C), their calculations predict occupation of the (12*k*)_Oh_ site to be dominant compared to (2*a*)_Oh_, and to increase from x = 0.5 to x = 1. Our results do show that the relative occupation of Al in the (12*k*)_Oh_ site increases with increasing Al content. However, the (2*a*)_Oh_ site remains the dominant substitution site for all compositions, in contrast to the calculations by V. Dixit et al. for such synthesis temperatures. According to their calculations, the probability of the Al^3+^ ion occupying the (4*f*)_Oh_, (4*f*)_Td_ or (4*e*)_BP_ sites is much lower compared to that of the other two sites. This is also what we observe here, both for the SG and the SSM series. The calculations by V. Dixit et al. in fact indicate the occupation of the (4*f*)_Oh_, (4*f*)_Td_ and (4*e*)_BP_ sites to be negligible, which is in accordance with our results for the SSM samples and with the SG sample with less Al content (SG x = 1). In this sample, the occupation fraction of Al in those three sites ((4*f*)_Oh_, (4*f*)_Td_ and (4*e*)_BP_) is so low, that 3*sigma is effectively equal to the occupation fraction. Therefore, Al occupation on those sites could be considered effectively zero for that sample, which has the same composition as that used by V. Dixit et al. for their calculations. As the concentration of Al increases, the occupation of the (4*f*)_Td_ remains negligible, but it increases slightly on the (4*f*)_Oh_ and (4*e*)_BP_ sites, reaching equal occupation in both sites at *x* = 3. The relative affinity of Al for the different sites and its variation with increasing substitution can be clearly evaluated when calculating the deviation from random occupation of Al on each site (Figure 8b). It is evident from Figure 8b that Al has a particular affinity for the (2*a*)_Oh_ and (12*k*)_Oh_ sites, and therefore the occupation of Al in those sites is higher compared to a random distribution. The three remaining sites, i.e. (4*f*)_Oh_, (4*f*)_Td_, and (4*e*)_BP_, are “avoided” by Al, and are thus being underpopulated with respect to a random occupation. However, as the Al content increases, the occupation of Al on the (2*a*)_Oh_ and (12*k*)_Oh_ sites is reduced, and the occupation of (4*f*)_Oh_ increases. These results clearly display how the relative affinity for the (4*f*)_Td_, and (4*e*)_BP_ sites (represented in red and gray respectively in Figure 8) stays unaffected (within the error) by the increase of total Al content in the structure, and the interplay of Al site occupation takes place between the remaining three sites, which are all octahedrally coordinated sites, with moment pointing up in (2*a*)_Oh_ and (12*k*)_Oh_ or down in (4*f*)_Oh_.

Our findings align with several previous studies on Al-substituted SrFe_12_O_19_, including those by Kazin et al.^23^ and Maltoni et al.,^42^ which used PXRD data to investigate the site occupation of Al within the structure. Similarly, our results are consistent with studies by Gorbachev et al.^26^ on Ca–Al-substituted SrFe_12_O_19_ and by Stingaciu et al.^31^ on the cosubstitution of Cr and Al into Ca-substituted Sr_0.67_Ca_0.33_Fe_9_Al_3‑x_Cr_*x*_O_19_. Collectively, these studies suggest that Al exhibits a clear site preference that remains relatively consistent, even with the cosubstitution of other elements. Additionally, Bilovol et al.^43^ conducted Mössbauer studies on Al-substituted BaFe_12_O_19_, concluding that Al substitution predominantly occurs in the 12*k*, 2*a*, and/or 4*f*_2_(Oh) sites. However, their findings differ slightly from those observed for SrFe_12‑*x*_Al*_x_* O_19_, particularly in the higher preference for Al occupation at the 12*k* site compared to the 2*a* site.

From the Rietveld refinements of the NPD patterns, the magnetic moments of the five different Fe^3+^ crystallographic sites were extracted. The refined absolute values of the moments for the SSM and SG samples as a function of refined Al^3+^ content are shown in Figure 10a,b, respectively, and numerical values are given in Table 2 for SG x = 2 and in equivalent tables in Supporting Information for the remaining samples.

**Figure 10:**
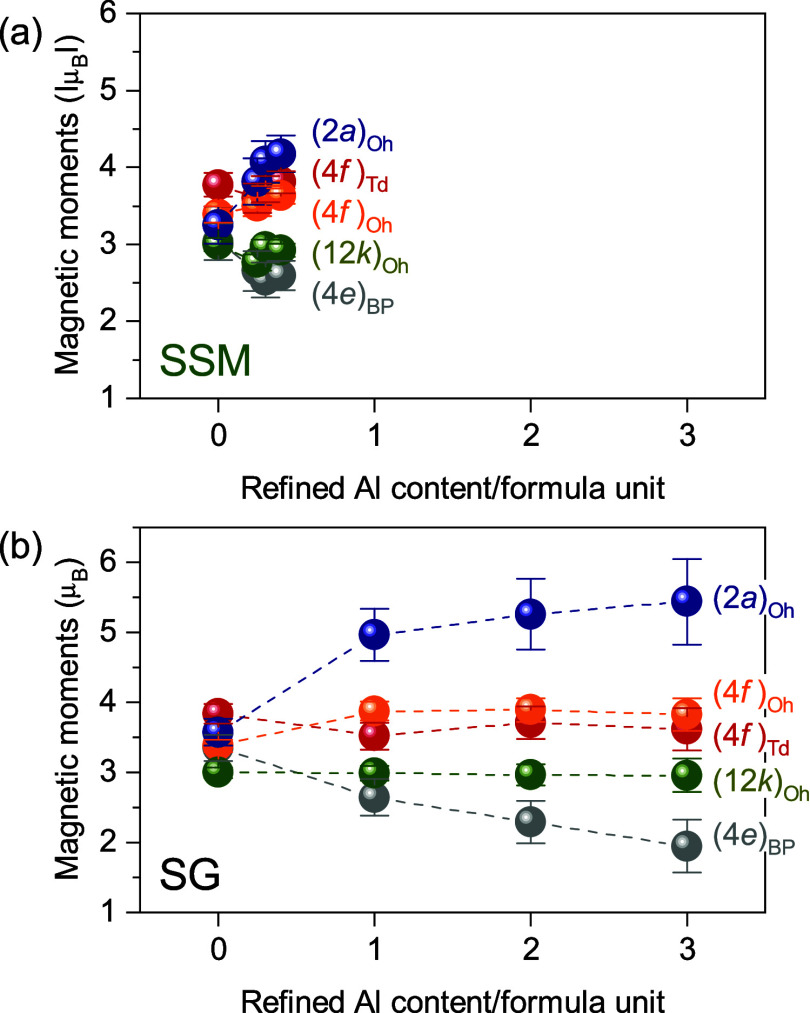
Refined magnetic moments of Fe^3+^ on the five different crystallographic Fe^3+^ sites with increasing refined aluminum content, for samples synthesized by (a) SSM and (b) SG methods. An equivalent figure plotted as a function of nominal Al content/formula unit can be found in Figure S16.

The refined magnetic moments of the SSM samples remain practically unchanged (within the uncertainty) for all samples throughout the series. These results confirm, once again, none or negligible Al substitution into the SrFe_12_O_19_ crystallites by the solid-salt-matrix synthesis method. In the SG samples, the magnetic moment of Fe^3+^ in three crystallographic sites (12*k*)_Oh_ (green), (4*f*)_Oh_ (orange) and (4*f*)_Td_ (red) remains unaffected by partial substitution of Al. However, the average magnetic moment of Fe^3+^ in the (2*a*)_Oh_ site (blue) increases, while it decreases in the (4*e*)_BP_ site (gray), showing a clear impact of the Al substitution on the atomic magnetic structure. As shown earlier (see Figure 8), Al has the highest affinity for the (2*a*)_Oh_ site (blue) and thus to maintain the long-range magnetic structure, a stronger moment may be required by the remaining Fe on the (2*a*)_Oh_ site. Although much more subtle, a slight increase in the magnetic moment of the (2*a*)_Oh_ site is also observed in the SSM series, where refinements indicated it to be the only site with Al substitution. In contrast, the bipyramidal (4*e*)_BP_ site (gray), where only moderate Al substitution is observed (see Figure 8), exhibits a decrease in magnetic moment that may be attributed to the lack of stabilizing moments (i.e., higher Al substitution) on the corner-sharing (12*k*)_Oh_ and (4*f*)_Td_ sites, through which superexchange interactions take place to give rise to the ferrimagnetic long-range structure.^44,45^ This site, (4*e*)_BP_, which is located in the atomic layer where a Sr atom replaces an oxygen atom, plays a key role in the hexaferrite structure, as the spin contribution from the iron atom in this site is the major contributor to the uniaxial anisotropy of the compound.^1,46^ However, due to the proximity of the Sr atom, the Fe^3+^ atom in the bipyramidal site has fewer Fe–O–Fe superexchange connections with neighboring Fe^3+^ atoms, and its magnetic moment is hence highly affected by modifications in the composition of the surrounding Fe sites. The electronic structure of the stoichiometric strontium hexaferrite was calculated by Fang et al.^44^ To understand the exchange interactions between Fe ions, they compare the partial density of states (DOS) of the Fe ions and total DOS for SrFe_12_O_19_ in a ferromagnetic state (all spins oriented in the same direction) with those of the ferrimagnetic structure described by Gorter et al.,^47^ where the 2*b*, 2*a* and 12*k* sites have their spin orientation antiparallel to that of the iron ions at the 4*f* sites. Notably, it is established that the hexaferrite adopts Gorter’s ferrimagnetic (rather than the ferromagnetic) structure. Their calculations conclude that is in fact the strong exchange interactions between the Fe ions at bipyramidal (which they consider as 2*b*) and 12*k* sites and those at the 4*f*_1_ and 4*f*_2_ sites that cause the instability of a ferromagnetic structure, and lead to the observed ferrimagnetic structure.

Additionally, in the studies by Grill et al. on the effect of diamagnetic substitutions in BaFe_12_O_19_,^48^ they conclude that that diamagnetic substitutions of Fe^3+^ ions which do not change the exchange parameters, cannot improve the hard magnetic properties of BaFe_12_O_19_ at room temperature, and that improvements to the calculations can potentially be made by substituting diamagnetic ions which can change the exchange parameters by significantly changing the anion lattice or the unit cell dimensions of the ferrite. As evident from our results, as well as previously reported studies, the substitution of Al in the structure does give rise to a change of the unit cell dimensions and an improvement of the hard magnetic properties. These studies therefore support the importance of the exchange interaction between the Fe sublattices, and in particular, the study by Fang et al. highlights the importance of exchange interaction between the 2*b* and 12*k* sites in the determination of the magnetic spin configuration of the compound. These conclusions therefore support the idea that Al substitution would have an impact on the exchange interactions, and that substitution of Al on the 12*k* site could influence the 2*b*(4*e*)-12*k* super exchange interaction and, consequently, the magnetic moment of the Fe ion in the bipyramidal site. Nonetheless, understanding how exactly it would influence the exchange interactions is not straightforward, given that, as Fang et al. state in their studies “the crystal splitting of the Fe ions at 2*b* sites is very complicated due to the trigonal bipyramidal coordination, as well as the super-exchange interactions between the Fe ion at 2*b* and those at 12*k*.” Our previous studies on SrFe_12_O_19_ nanoplatelets also showed how the magnetic moment in the 4*e* site was highly affected by extreme reduction in the platelet thickness.^9^ This also explains that the SSM samples consistently has the lowest magnetic moment on the (4*e*)_BP_ site as these crystallites are only ∼23 nm thick.

The intrinsic crystallographic magnetization for each sample can be extracted from the Rietveld refinements of the NPD data taking into account the refined magnetic moment of Fe^3+^ per site, the multiplicity of each site and the site occupation fraction of Fe on each site. A comparison between the measured (*M*_s_, *M*_r_) and refined (*M*_NPD_) magnetization values of the SSM and SG series is shown in Figure 11.

**Figure 11:**
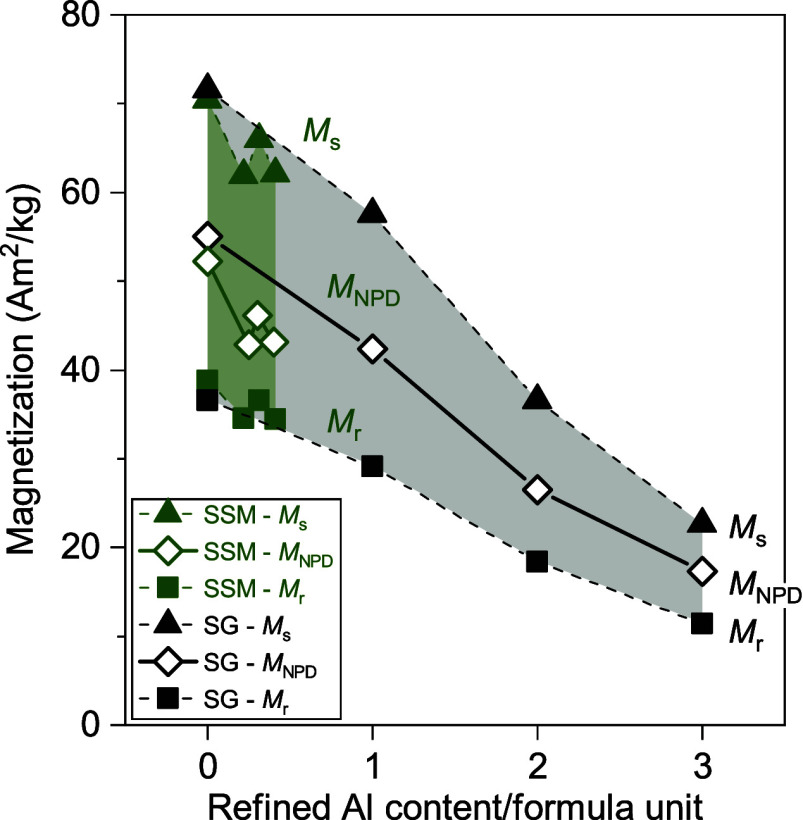
Measured saturation magnetization (*M*_s_), remanent magnetization (*M*_r_) and calculated magnetization from the refined magnetic moments of the NPD data (*M*_NPD_) for both, the SG and SSM series, as a function of refined Al substitution. Error bars within the size of the symbols. An equivalent figure plotted as a function of nominal Al content/formula unit can be found in Figure S17.

For the SSM samples (green in Figure 11), a slight decrease is observed in both the measured and refined magnetization values for aluminum substitution. However, very similar values are obtained for nominal Al values of X = 1 – 3, corresponding to refined values of 0.25, 0.40, and 0.30. This is consistent with the Rietveld results, indicating that a very small amount of Al is indeed substituted in the structure of the SSM samples, but it is very minor, it does not correspond to the nominal Al content, and it does not vary much regardless of an increasing amount of Al being added during the synthesis process. As confirmed by the agreement in the trend between the measured and refined magnetization values (Figure 11), the small decrease in the attained magnetization can be explained by the minor amount of nonmagnetic Al^3+^ cations effectively substituted in the (2*a*)_Oh_ site.

For the SG series (black in Figure 11), the calculated magnetization extracted from the Rietveld refinement of the NPD data shows a continuous decrease in saturation magnetization with increasing Al content, which is consistent with the observed trend in the measured magnetic data. The reduction in the total magnetization of the sample with increasing Al content is due to the nonmagnetic nature of Al^3+^ cations and their specific site occupation within the crystal structure.

As shown in Figure 11, the *M*_NPD_ value is found to be precisely between that of the saturation and remanent magnetization values obtained from VSM measurements, as expected. The measured saturation *M*_s_ should be equivalent to the refined magnetization, if NPD data were collected at 0 K. Here, however, the refined magnetization is obtained from room temperature NPD data. At room temperature, and without any applied magnetic field, the atomic magnetic dipolar moment will precess due to thermal fluctuations. This will reduce the projection of the magnetic moment vectors onto the *c*-axis with respect to the fully aligned moments and therefore reduce the “observed” scattering from the atomic magnetic dipolar moments.

The excellent agreement between refined and measured magnetization values validates the results obtained in the NPD refinements, which indicate a preferred site occupation of the Al^3+^ cations in the (2*a*)_Oh_ and (12*k*)_Oh_ Wyckoff sites, followed by (4*e*)_PB_ and (4*f*)_Oh_. Given that the three most substituted sites correspond to sites with the magnetic moment pointing up (main magnetization direction), substitution of Fe^3+^ by the nonmagnetic Al^3+^ onto those sites explains the observed reduction in the intrinsic magnetization of the compounds. The increase in coercivity can be explained by (and be a consequence of) the reduction in the saturation magnetization, if it is assumed that the magnetocrystalline anisotropy (*K*_1_) is the same for all samples, given that the coercivity is related to the saturation magnetization by the relation *H*_c_ = 2*K*_1_/*M*_s_.

The key factor driving the effective substitution of Al in the SG series compared to the AC and SSM, where lack or negligible Al is substituted, could potentially be the higher synthesis temperature employed in the SG (925 °C) compared to the AC (240 °C) and SSM (790 °C). This is particularly evident when comparing the SG and AC syntheses, as the AC method uses a significantly lower temperature (685 °C lower) than the SG. Although the temperature difference between the SG and SSM syntheses is smaller (135 °C), the lower temperature in the SSM process still suggests that temperature plays an important role in Al substitution. However, temperature may not be the only factor influencing Al incorporation. Other variables, such as the intermediate species formed before the high-temperature treatment, could also have a significant impact. In the AC synthesis, Al most likely remains in solution as a hydroxide, given that it neither precipitates as a secondary phase nor integrates into the hexaferrite structure. It is possible that higher temperatures might promote the precipitation of these stable dissolved species. In the SSM synthesis, which employs temperatures closer to the SG method, Fe and Sr are introduced as chlorides rather than nitrates, while Al­(NO_3_)_3_ is used as the source of Al. The chlorides, which are essential for the formation of the salt matrix, could potentially inhibit the incorporation of Al into the structure, leaving most of the Al in the salt matrix, which is washed away.

## Conclusions

Partial substitution of Al into the SrFe_12_O_19_ structure was attempted by three synthesis methods, namely autoclave (AC), citrate sol–gel (SG) and solid-salt-matrix (SSM) synthesis. While no (or negligible) effective Al substitution was observed in the AC or SSM samples, successful Al substitution was attained in the SG samples, leading to a decrease of the unit cell axes and an increase of crystallite size with increasing Al content. Combined Rietveld refinements of NPD and PXRD data revealed that the nonmagnetic aluminum atoms predominantly occupy the sites with the magnetic moment pointing up; i.e. (2*a*)_Oh_, (12*k*)_Oh_ and (4*e*)_BP_, but as the Al content increases, the (4*f*)_Oh_ site whose magnetic moment is pointing down, also becomes partially populated by Al. As a consequence of Al predominantly occupying sites with the magnetic moment pointing up, the net magnetic moment of the compound is diminished. The macroscopic magnetization of the samples is reduced with increasing Al substitution, which is in excellent agreement with the refined magnetic moments and Al site occupation fractions, as well as with previously reported theoretical calculations. Notably, very high coercivity values are obtained by Al substitution, reaching a maximum of 830(21) kA/m (10.4 kOe) for SrFe_9_Al_3_O_19_. Not only does this value exceed that of the unsubstituted SrFe_12_O_19_ by ∼71% but is comparable to the coercivity of Nd_2_Fe_17_B magnets.

## Supplementary Material



## Notes

Additional information on refined parameters and implemented constraints. Rietveld refinement plots of PXRD and NPD data for all samples. Tables containing all refined parameters of all refined phases for all samples. Plot of refined site occupation fraction of Al for SSM samples. Refined magnetic moments of SSM and SG samples as a function of nominal Al content/formula unit. Refined magnetic moments and measured magnetization values of SSM and SG samples plotted as a function of nominal Al content/formula unit (PDF)
