# Supplementary material for: Synthesis, Crystal Structure, Site Occupancy, and Magnetic Properties of Aluminum-Substituted M‑Type Sr Hexaferrite SrFe12‑xAl x O19 Nanoparticles
Source: Chem Mater. 2025 Jan 20;37(3):884–96. doi: 10.1021/acs.chemmater.4c02205 (PMC12127982; doi:10.1021/acs.chemmater.4c02205)
Supplement: Supplementary file 1 [file cm4c02205_si_001.pdf]

Electronic Supporting Information for:

Synthesis, crystal structure, site occupancy and magnetic properties of aluminum substituted M-type Sr hexaferrite $SrFe_{12-x}Al_xO_{19}$ nanoparticles.

Matilde Saura-Múzquiz,[a†*] Anna Zink Eikeland,[b†] Marian Stingaciu,[b] Henrik Lyder Andersen,[c] Maxim Avdeev,[d] and Mogens Christensen[b]

[a] Departamento de Física de Materiales, Faculty of Physics, Universidad Complutense de Madrid, Plaza de Ciencias 1, Ciudad Universitaria, Madrid 28040, Spain
[b] Center for Materials Crystallography, Department of Chemistry and iNANO, Aarhus University, 8000 Aarhus C, Denmark
[c] Instituto de Ciencia de Materiales de Madrid (ICMM), CSIC, Madrid 28049, Spain
[d] Australian Nuclear Science and Technology Organisation (ANSTO), New Illawarra Road, Lucas Heights, NSW 2234, Australia.
[†] Equally contributing authors

** Email: matsaura@ucm.es*

Contents

Additional information on refined parameters and constraints 3
Rietveld refinements of PXRD data of AC samples 4
  AC x=0 ($SrFe_{12}O_{19}$) 4
    Refinement profile 4
    Refined parameters 5
  AC x=0.5 ($SrFe_{11.5}Al_{0.5}O_{19}$) 6
    Refinement profile 6
    Refined parameters 7
  AC x=1 ($SrFe_{11}AlO_{19}$) 8
    Refinement profile 8
    Refined parameters 8
  AC x=2 ($SrFe_{10}Al_2O_{19}$) 9
    Refinement profile 9
    Refined parameters 9
  AC x=2.5 ($SrFe_{9.5}Al_{2.5}O_{19}$) 10
    Refinement profile 10
    Refined parameters 10
  AC x=3 ($SrFe_9Al_3O_{19}$) 11
    Refinement profile 11
    Refined parameters 11
Combined Rietveld refinements of PXRD and NPD data of SSM samples 12
  SSM x=0 ($SrFe_{12}O_{19}$) 12
    Refinement profile 12

Refined parameters 13
SSM x=1 ($SrFe_{11}AlO_{19}$) 14
Refinement profile 14
Refined parameters 15
SSM x=2 ($SrFe_{10}Al_2O_{19}$) 16
Refinement profile 16
Refined parameters 17
SSM x=3 ($SrFe_{19}Al_3O_{19}$) 18
Refinement profile 18
Refined parameters 19
Site Occupation Fraction of Al cations in the SSM samples 20
Combined Rietveld refinements of PXRD and NPD data of SG samples 21
SG x=0 ($SrFe_{12}O_{19}$) 21
Refinement profile 21
Refined parameters 22
SG x=1 ($SrFe_{11}AlO_{19}$) 23
Refinement profile 23
Refined parameters 24
SG x=2 ($SrFe_{10}Al_2O_{19}$) 25
Refinement profile 25
Refined parameters 26
SG x=3 ($SrFe_9Al_3O_{19}$) 27
Refinement profile 27
Refined parameters 28
Refined magnetic moments as function of nominal Al content 29
Refined and measured magnetization as function of nominal Al content 30

Additional information on refined parameters and constraints

The general refinement strategy is described in the "Characterization" section of the main manuscript.
Here, additional information is provided in relation to constraints implemented in the refinements. All refined parameters and esd's are given in the corresponding tables of this document.

For samples where PXRD and NPD data were collected (i.e. SSM and SG), the following constraints were implemented in the refinements:

- The isotropic displacement parameters (B_{iso}) were refined, constraining the B_{iso} values to be equal for the same atom type (i.e. Sr/Fe/O) and for different atom types occupying the same sites (i.e. Fe and Al).
- The unit cell parameters were constrained to be equal in both PXRD and NPD patterns.
- The refinable Y and Sz parameters of both patterns (PXRD and NPD) were constrained to return the same crystallite sizes, taking into consideration the relationship between the refinable parameters and the obtained crystallite sizes, and the difference in wavelength of both patterns. That is, Y was not simply constrained to be equal in both patterns, but rather it was constrained according to the following relation:

  In FullProf:

$$Crystallite\ size = \frac{1}{Y + FS} * 100$$

$$FS = S_z \cdot cos\theta$$

$$Y(\text{Å}^{-1}) = Y_{Refined} \cdot \frac{\pi}{180} \cdot \frac{\pi}{2} \cdot \frac{1}{\lambda(\text{Å})} \cdot 1000$$

  If the refined crystallite size from PXRD and NPD patterns must be the same, then:

$$(Y + FS)_{NPD} = (Y + FS)_{PXRD}$$

  Therefore, if $FS_{NPD} = FS_{PXRD}$:

$$S_{z(NPD)} = S_{z(PXRD)}$$

  However, if $Y_{NPD}(\text{Å}^{-1}) = Y_{PXRD}(\text{Å}^{-1})$, the refined Y values of NPD and PXRD patterns have the following relation:

$$Y_{NPD} \cdot \frac{1}{\lambda_{NPD}} = Y_{PXRD} \cdot \frac{1}{\lambda_{PXRD}}$$

$$Y_{NPD} = Y_{PXRD} \cdot \frac{\lambda_{NPD}}{\lambda_{PXRD}} \quad ; \quad Y_{PXRD} = Y_{NPD} \cdot \frac{\lambda_{PXRD}}{\lambda_{NPD}},$$

  Therefore, the S_z parameters were refined to be equal in both patterns, and the Y parameters were refined according to the ratio of the corresponding wavelengths, calculating the corresponding refinable value of each pattern, as well as allowing it to refine according to wavelength ratio. That is, if $\lambda_{NPD} = 2.43973$ Å and $\lambda_{PXRD} = 1.78919$ Å, then ${}^{\lambda_{PXRD}}/_{\lambda_{NPD}} = 0.73335$. Therefore:

$$Y_{PXRD} = Y_{NPD} \cdot 0.73335$$

  And while Y_{NPD} will be allowed to refine for a factor 1 in each refinement cycle, Y_{PXRD} will be allowed to refine a factor 0.73335 in each cycle, ensuring that the relationship between Y_{PXRD} and Y_{NPD} remains unchanged.

In addition to this, the constraints and restraints carried out for elucidating the Al content in the SG and SSM samples are explained in detail in the manuscript, in the section "*Aluminum site occupancy and magnetic structure– Combined NPD and PXRD Rietveld refinements*" of Results and discussion.

Rietveld refinements of PXRD data of AC samples

AC x=0 ($SrFe_{12}O_{19}$)

Refinement profile

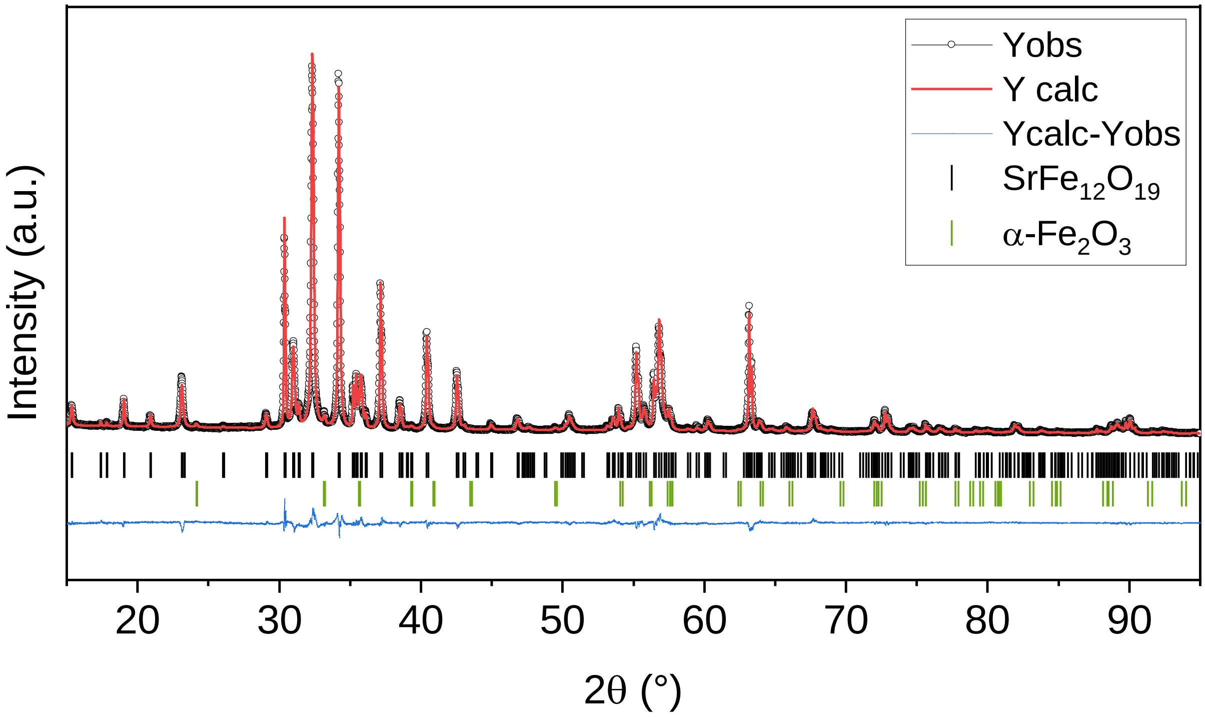

Figure S1

Refined parameters

Phase N. 1: $SrFe_{12}O_{19}$
Space group: P 63/m m c (194); general multiplicity: 24
Phase fraction: 98.2(3)% Bragg R-factor: 4.60 RF-factor: 4.60 <- Pat#1
Composition: Sr2 Fe24 O38 Theoretical density: 5.1 g/cm3
a = 5.88245(2)Å, b = 5.88245(2)Å, c = 23.0744(1)Å, α = 90.00° β = 90.00° γ = 120.000°
Y= 0.037(4)
S_z= 1.79(1)

Atom & site	x	y	z	B_{iso} (Å^2)	Occ.	Mult.	Compos.	Fraction
Sr (2*d*)	0.3333	0.6667	0.75	2.99(4)	0.08333	2	2	1.0
Fe1 (2*a*)$_{Oh}$	0	0	0	3.08(7)	0.08333	2	2	1.0
Fe2 (12*k*)$_{Oh}$	0.1684(2)	0.3368(5)	-0.10914(3)	2.75(2)	0.5	12	12	1.0
Fe3 (4*f*)$_{Oh}$	0.3333	0.6667	0.18932(6)	2.84(4)	0.16667	4	4	1.0
Fe4 (4*f*)$_{Td}$	0.3333	0.6667	0.02700(7)	2.90(5)	0.16667	4	4	1.0
Fe5 (4*e*)$_{BP}$	0	0	0.2584(2)	1.99(10)	0.08333	4	2	0.5
O1 (*4e*)	0	0	0.1508(2)	2.34(4)	0.16667	4	4	1.0
O2 (*4f*)	0.3333	0.6667	-0.0556(2)	2.34(4)	0.16667	4	4	1.0
O3 (*6h*)	0.1845(10)	0.369(2)	0.25	2.34(4)	0.25	6	6	1.0
O4 (*12k*)	0.1599(7)	0.320(1)	0.0532(1)	2.34(4)	0.5	12	12	1.0
O5 (*12k*)	0.501(1)	0.002(2)	0.1493(1)	2.34(4)	0.5	12	12	1.0

Phase N. 2: α-Fe_2O_3
Space group: R -3 c (167); general multiplicity: 36
Phase fraction: 1.85(9)% Bragg R-factor: 21.30 RF-factor: 10.70 <- Pat#1
Composition: Fe12 O18 Theoretical density: 5.27 g/cm3
a = 5.0372(4)Å, b = 5.0372(4)Å, c = 13.743(1)Å, α = 90.00° β = 90.00° γ = 120.000°
Y = 0.112(8)

Atom & site	x	y	z	B_{iso} (Å^2)	Occ.	Mult.	Compos.	Fraction
Fe (12*c*)	0	0	0.3553	3.7(4)	0.33333	12	12	1.0
O (18*e*)	0.3059	0	0.25	3.8(10)	0.5	18	18	1.0

AC x=0.5 ($SrFe_{11.5}Al_{0.5}O_{19}$)

Refinement profile

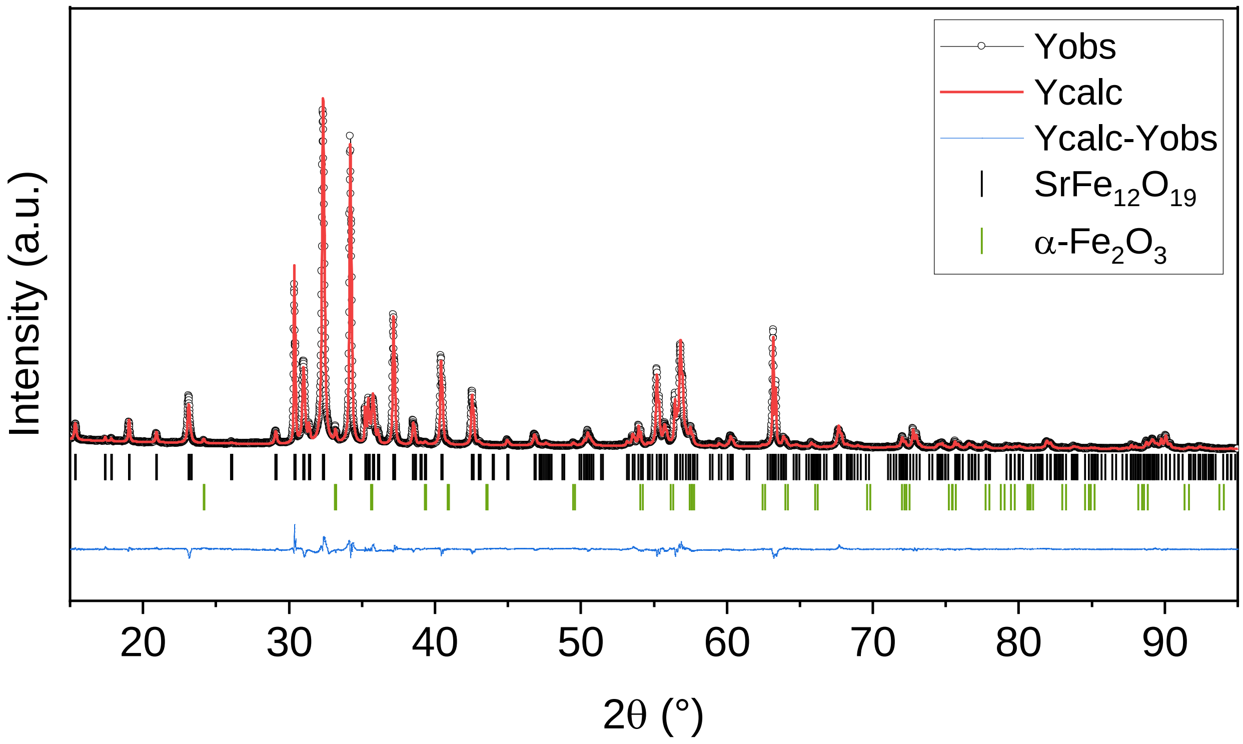

Figure S2

Refined parameters

Phase N. 1: $SrFe_{12}O_{19}$

Space group: P 63/m m c (194); general multiplicity: 24

Phase fraction: 97.8(3)% Bragg R-factor: 4.95 RF-factor: 4.53 <- Pat#1

Composition: Sr2 Fe24 O38 Theoretical density: 5.101 g/cm3

a = 5.88212(2)Å, b = 5.88212(2)Å, c = 23.0723(1)Å, α = 90.00° β = 90.00° γ = 120.000°

Y= 0.0340(4)

S_z= 1.73(1)

**Atom & site**	**x**	**y**	**z**	B_{iso} (Å^2)	**Occ.**	**Mult.**	**Compos.**	**Fraction**
Sr (2*d*)	0.3333	0.6667	0.75	2.76(4)	0.08333	2	2	1.0
Fe1 $(2a)_{Oh}$	0	0	0	2.94(6)	0.08333	2	2	1.0
Fe2 $(12k)_{Oh}$	0.1680(2)	0.3361(5)	-0.10913(3)	2.40(2)	0.5	12	12	1.0
Fe3 $(4f)_{Oh}$	0.3333	0.6667	0.18950(6)	2.60(4)	0.16667	4	4	1.0
Fe4 $(4f)_{Td}$	0.3333	0.6667	0.02680(7)	2.56(5)	0.16667	4	4	1.0
Fe5 $(4e)_{BP}$	0	0	0.2581(2)	1.72(9)	0.08333	4	2	0.5
O1 (*4e*)	0	0	0.1526(2)	1.80(4)	0.16667	4	4	1.0
O2 (*4f*)	0.3333	0.6667	-0.0560(2)	1.80(4)	0.16667	4	4	1.0
O3 (*6h*)	0.1823(10)	0.365(2)	0.25	1.80(4)	0.25	6	6	1.0
O4 (*12k*)	0.1609(7)	0.322(1)	0.05294(9)	1.80(4)	0.5	12	12	1.0
O5 (*12k*)	0.5011(10)	0.002(2)	0.1497(1)	1.80(4)	0.5	12	12	1.0

Phase N. 2: α-Fe_2O_3

Space group: R -3 c (167); general multiplicity: 36

Phase fraction: 2.21(9)% Bragg R-factor: 19.90 RF-factor: 10.20 <- Pat#1

Composition: Fe12 O18 Theoretical density: 5.272 g/cm3

a = 5.0356(2)Å, b = 5.0356(2)Å, c = 13.7443(9)Å, α = 90.00° β = 90.00° γ = 120.000°

Y= 0.085(5)

**Atom & site**	**x**	**y**	**z**	B_{iso} (Å^2)	**Occ.**	**Mult.**	**Compos.**	**Fraction**
Fe (12*c*)	0	0	0.3553	3.5(3)	0.33333	12	12	1.0
O (18*e*)	0.3059	0	0.25	3.9(8)	0.5	18	18	1.0

AC x=1 ($SrFe_{11}AlO_{19}$)

Refinement profile

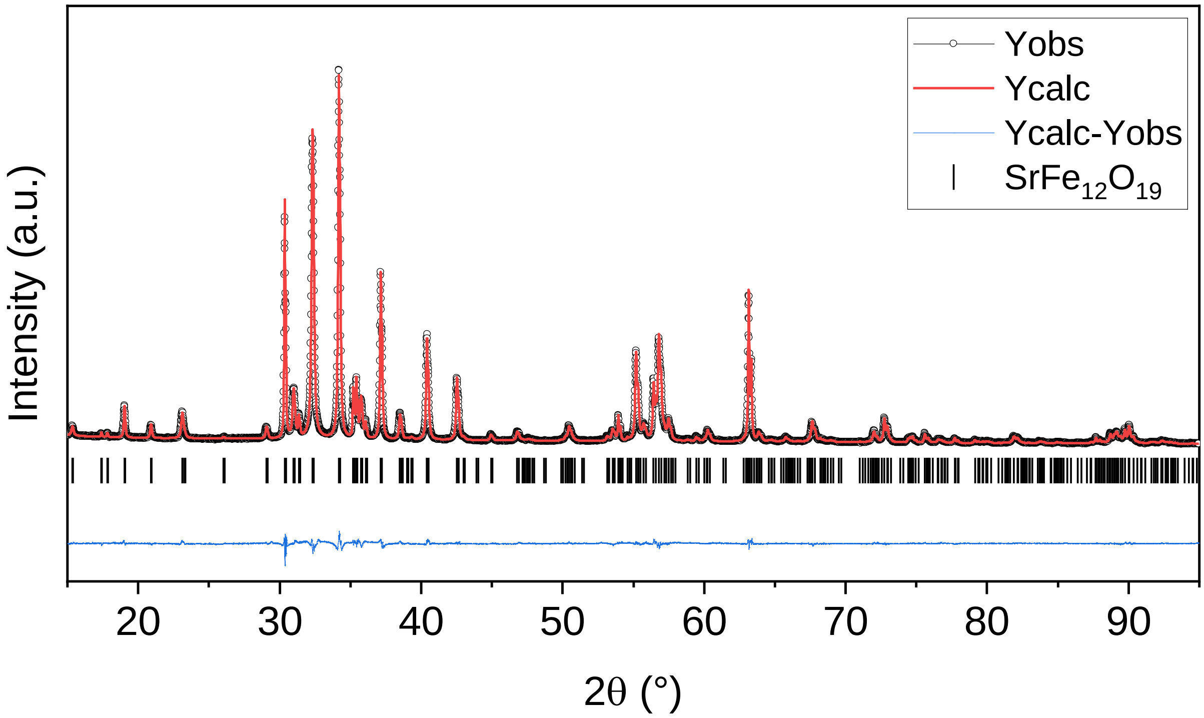

Figure S3

Refined parameters

Phase N. 1: $SrFe_{12}O_{19}$

Space group: P 63/m m c (194); general multiplicity: 24

Phase fraction: 100.0(3)% Bragg R-factor: 2.78 RF-factor: 3.26 <- Pat#1

Composition: Sr2 Fe24 O38 Theoretical density: 5.098 g/cm3

a = 5.88349(2)Å, b = 5.88349(2)Å, c = 23.0762(1)Å, α = 90.00° β = 90.00° γ = 120.000°

Y= 0.037(3)

S_z= 1.74(1)

Atom & site	x	y	z	B_{iso} (Å^2)	Occ.	Mult.	Compos.	Fraction
Sr (2*d*)	0.3333	0.6667	0.75	2.49(4)	0.08333	2	2	1.0
Fe1 (2*a*)$_{Oh}$	0	0	0	2.43(5)	0.08333	2	2	1.0
Fe2 (12*k*)$_{Oh}$	0.1683(2)	0.3367(4)	-0.10898(3)	2.10(2)	0.5	12	12	1.0
Fe3 (4*f*)$_{Oh}$	0.3333	0.6667	0.18967(5)	2.02(4)	0.16667	4	4	1.0
Fe4 (4*f*)$_{Td}$	0.3333	0.6667	0.02688(6)	2.55(4)	0.16667	4	4	1.0
Fe5 (4*e*)$_{BP}$	0	0	0.2576(3)	2.33(8)	0.08333	4	2	0.5
O1 (4*e*)	0	0	0.1515(2)	1.54(4)	0.16667	4	4	1.0
O2 (4*f*)	0.3333	0.6667	-0.0558(2)	1.54(4)	0.16667	4	4	1.0
O3 (6*h*)	0.1839(8)	0.368(2)	0.25	1.54(4)	0.25	6	6	1.0
O4 (12*k*)	0.1600(6)	0.320(1)	0.05254(8)	1.54(4)	0.5	12	12	1.0
O5 (12*k*)	0.5037(7)	0.007(1)	0.1520(1)	1.54(4)	0.5	12	12	1.0

AC x=2 ($SrFe_{10}Al_2O_{19}$)

Refinement profile

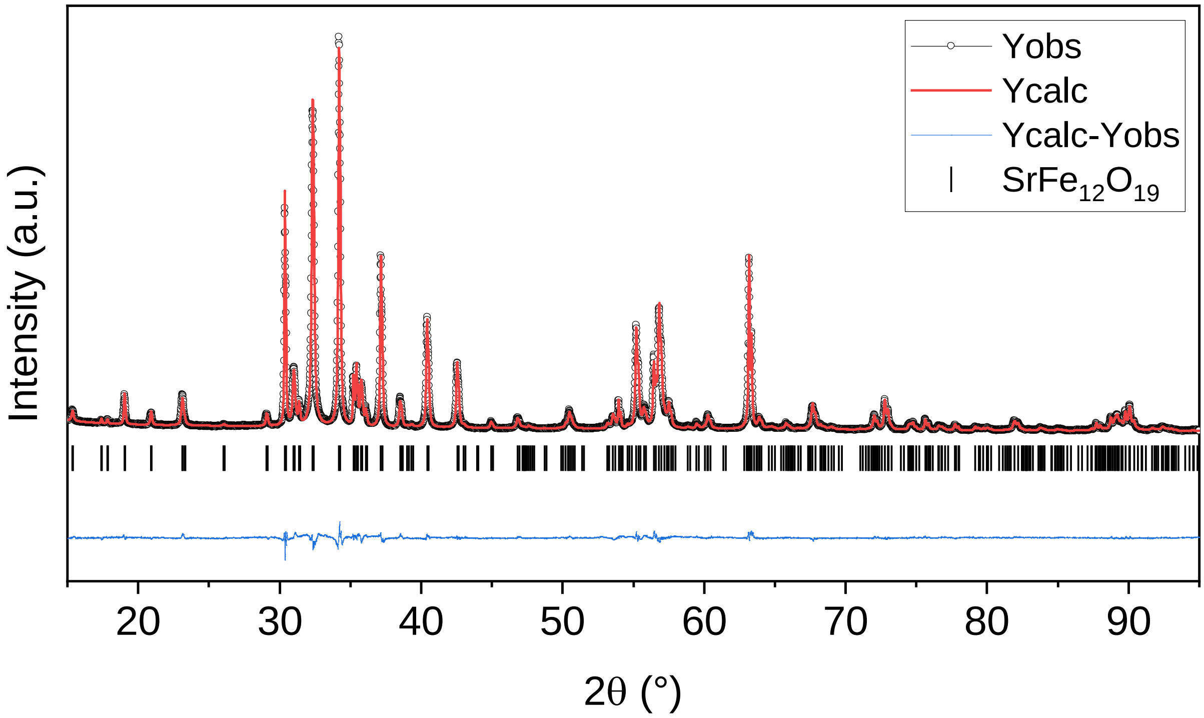

Figure S4

Refined parameters

Phase N. 1: $SrFe_{12}O_{19}$

Space group: P 63/m m c (194); general multiplicity: 24

Phase fraction: 100.0(3)% Bragg R-factor: 3.34 RF-factor: 3.85 <- Pat#1

Composition: Sr2 Fe24 O38 Theoretical density: 5.103 g/cm3

a = 5.88166(2)Å, b = 5.88166(2)Å, c = 23.0685(1)Å, α = 90.00° β = 90.00° γ = 120.000°

Y= 0.0417(3)

S_z= 1.73(1)

Atom & site	x	y	z	B_{iso} (Å^2)	Occ.	Mult.	Compos.	Fraction
Sr (2*d*)	0.3333	0.6667	0.75	1.62(4)	0.08333	2	2	1.0
Fe1 (2*a*)$_{Oh}$	0	0	0	1.59(6)	0.08333	2	2	1.0
Fe2 (12*k*)$_{Oh}$	0.1682(2)	0.3364(4)	-0.10903(3)	1.31(2)	0.5	12	12	1.0
Fe3 (4*f*)$_{Oh}$	0.3333	0.6667	0.18970(6)	1.24(4)	0.16667	4	4	1.0
Fe4 (4*f*)$_{Td}$	0.3333	0.6667	0.02688(7)	1.64(4)	0.16667	4	4	1.0
Fe5 (4*e*)$_{BP}$	0	0	0.2564(3)	1.41(9)	0.08333	4	2	0.5
O1 (4*e*)	0	0	0.1514(2)	0.70(4)	0.16667	4	4	1.0
O2 (4*f*)	0.3333	0.6667	-0.0557(2)	0.70(4)	0.16667	4	4	1.0
O3 (6*h*)	0.1842(9)	0.368(2)	0.25	0.70(4)	0.25	6	6	1.0
O4 (12*k*)	0.1603(6)	0.321(1)	0.05263(9)	0.70(4)	0.5	12	12	1.0
O5 (12*k*)	0.5037(8)	0.007(2)	0.1511(1)	0.70(4)	0.5	12	12	1.0

AC x=2.5 ($SrFe_{9.5}Al_{2.5}O_{19}$)

Refinement profile

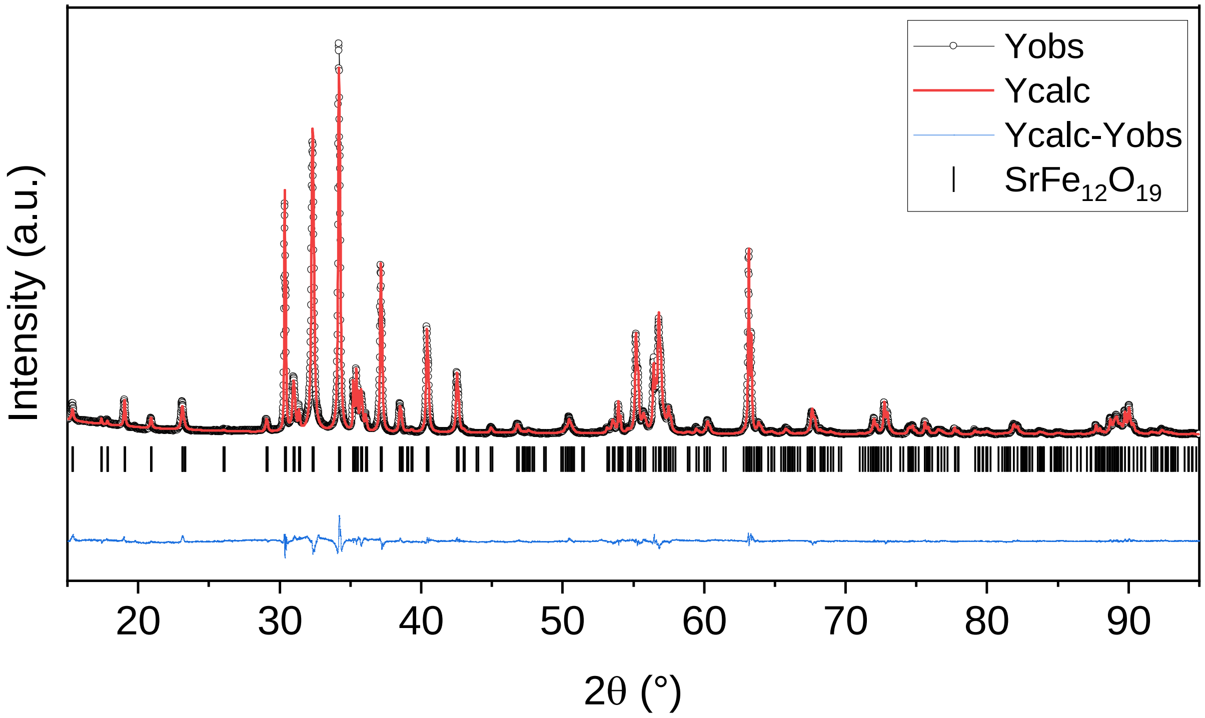

Figure S5

Refined parameters

Phase N. 1: $SrFe_{12}O_{19}$

Space group: P 63/m m c (194); general multiplicity: 24

Phase fraction: 100.0(3)% Bragg R-factor: 5.00 RF-factor: 4.62 <- Pat#1

Composition: Sr2 Fe24 O38 Theoretical density: 5.097 g/cm3

a = 5.88338(2)Å, b = 5.88338(2)Å, c = 23.0805(1)Å, α = 90.00° β = 90.00° γ = 120.000°

Y= 0.0383(3)

S_z= 1.90(1)

Atom & site	x	y	z	B_{iso} (Å^2)	Occ.	Mult.	Compos.	Fraction
Sr (2*d*)	0.3333	0.6667	0.75	1.35(4)	0.08333	2	2	1.0
Fe1 (2*a*)$_{Oh}$	0	0	0	1.15(6)	0.08333	2	2	1.0
Fe2 (12*k*)$_{Oh}$	0.1684(2)	0.3368(5)	-0.10931(3)	0.97(2)	0.5	12	12	1.0
Fe3 (4*f*)$_{Oh}$	0.3333	0.6667	0.18901(6)	0.86(4)	0.16667	4	4	1.0
Fe4 (4*f*)$_{Td}$	0.3333	0.6667	0.02711(7)	0.88(4)	0.16667	4	4	1.0
Fe5 (4*e*)$_{BP}$	0	0	0.2579(3)	0.46(8)	0.08333	4	2	0.5
O1 (4*e*)	0	0	0.1514(2)	0.64(4)	0.16667	4	4	1.0
O2 (4*f*)	0.3333	0.6667	-0.0551(2)	0.64(4)	0.16667	4	4	1.0
O3 (6*h*)	0.181(1)	0.362(2)	0.25	0.64(4)	0.25	6	6	1.0
O4 (12*k*)	0.1590(8)	0.318(2)	0.0533(1)	0.64(4)	0.5	12	12	1.0
O5 (12*k*)	0.502(1)	0.003(2)	0.1506(1)	0.64(4)	0.5	12	12	1.0

AC x=3 ($SrFe_9Al_3O_{19}$)

Refinement profile

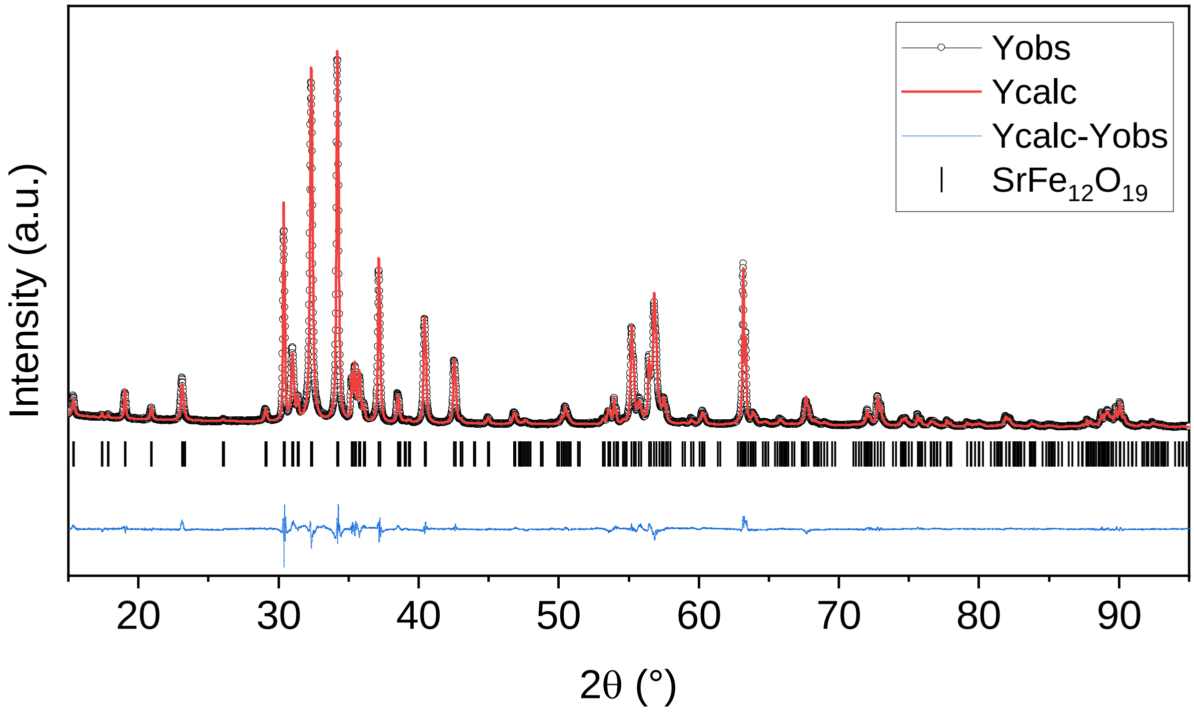

Figure S6

Refined parameters

Phase N. 1: $SrFe_{12}O_{19}$

Space group: P 63/m m c (194); general multiplicity: 24

Phase fraction: 100.0(3)% Bragg R-factor: 3.70 RF-factor: 3.64 <- Pat#1

Composition: Sr2 Fe24 O38 Theoretical density: 5.101 g/cm3

a = 5.88186(2)Å, b = 5.88186(2)Å, c = 23.0728(2)Å, α = 90.00° β = 90.00° γ = 120.000°

Y= 0.0528(4)

S_z= 1.73(2)

**Atom & site**	**x**	**y**	**z**	B_{iso} (Å^2)	**Occ.**	**Mult.**	**Compos.**	**Fraction**
Sr (2*d*)	0.3333	0.6667	0.75	1.85(5)	0.08333	2	2	1.0
Fe1 (2*a*)$_{Oh}$	0	0	0	1.81(7)	0.08333	2	2	1.0
Fe2 (12*k*)$_{Oh}$	0.1681(2)	0.3361(5)	-0.10909(3)	1.44(2)	0.5	12	12	1.0
Fe3 (4*f*)$_{Oh}$	0.3333	0.6667	0.18961(7)	1.47(5)	0.16667	4	4	1.0
Fe4 (4*f*)$_{Td}$	0.3333	0.6667	0.02735(8)	1.88(5)	0.16667	4	4	1.0
Fe5 (4*e*)$_{BP}$	0	0	0.2563(4)	1.5(1)	0.08333	4	2	0.5
O1 (4*e*)	0	0	0.1507(2)	0.80(4)	0.16667	4	4	1.0
O2 (4*f*)	0.3333	0.6667	-0.0564(2)	0.80(4)	0.16667	4	4	1.0
O3 (6*h*)	0.184(1)	0.369(2)	0.25	0.80(4)	0.25	6	6	1.0
O4 (12*k*)	0.1602(8)	0.320(2)	0.0532(1)	0.80(4)	0.5	12	12	1.0
O5 (12*k*)	0.503(1)	0.006(2)	0.1489(1)	0.80(4)	0.5	12	12	1.0

Combined Rietveld refinements of PXRD and NPD data of SSM samples

SSM x=0 ($SrFe_{12}O_{19}$)

Refinement profile

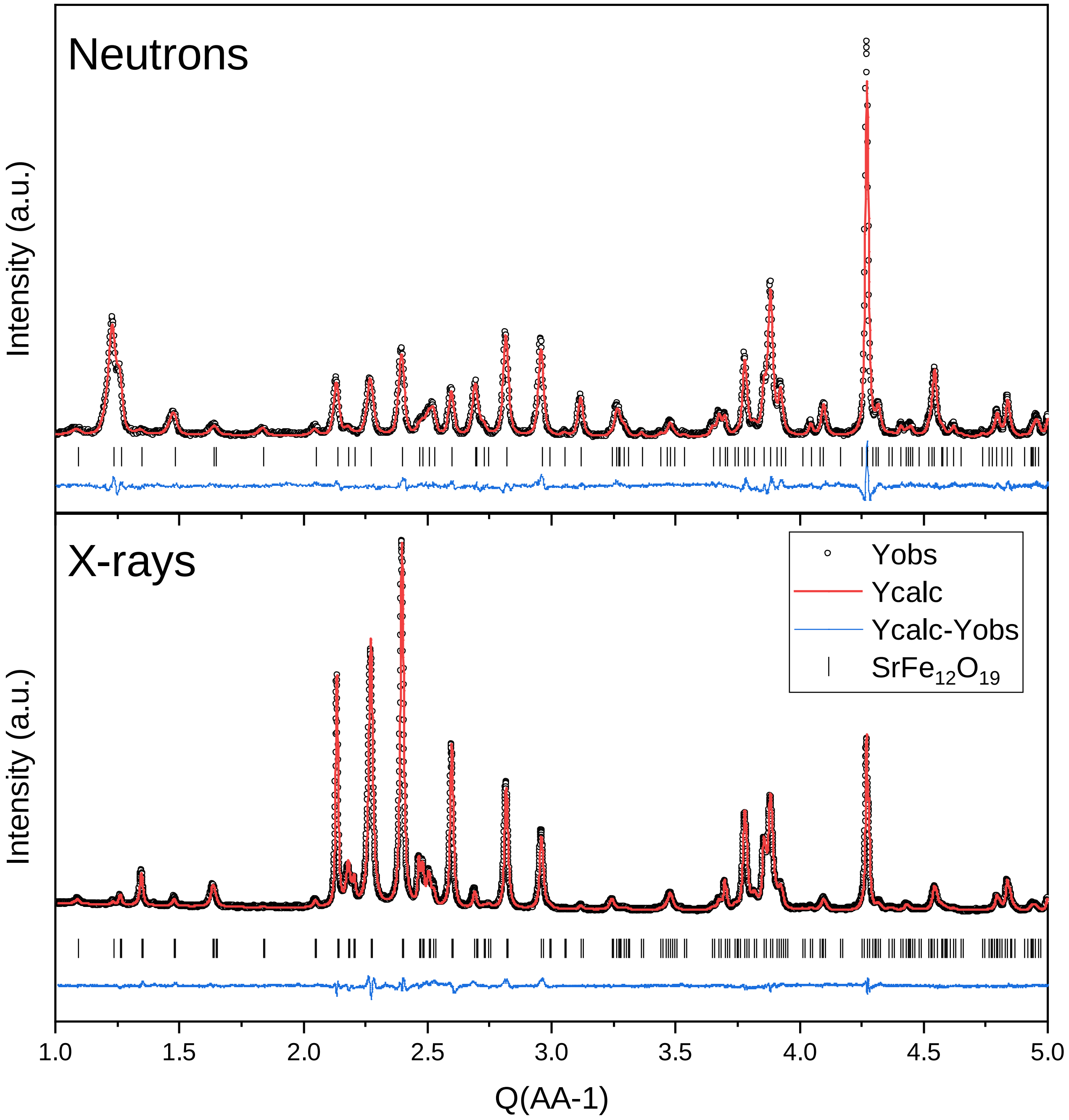

Figure S7

Refined parameters

Phase N. 1: $SrFe_{12}O_{19}$
Space group: P 63/m m c (194); general multiplicity: 24
Phase fraction: 100.0(8)% Bragg R-factor: 5.99 RF-factor: 4.25 <- Pat#1
Phase fraction: 100.0(3)% Bragg R-factor: 2.35 RF-factor: 3.25 <- Pat#2
Composition: Sr2 Fe24 O38 Theoretical density: 5.096 g/cm3
a = 5.88637(2)Å, b = 5.88637(2)Å, c = 23.0590(1)Å, α = 90.00° β = 90.00° γ = 120.000°
Y= 0.1483(5)
S_z= 2.54(2)

**Atom & site**	**x**	**y**	**z**	**B_{iso} (Å^2)**	**Occ.**	**Mult.**	**Compos.**	**Fraction**	**R_z (μ_B)**
Sr (2*d*)	0.3333	0.6667	0.75	2.00(4)	0.08333	2	2	1.0	
Fe1 (2*a*)$_{Oh}$	0	0	0	1.53(2)	0.08333	2	2	1.0	3.3(2)
Fe2 (12*k*)$_{Oh}$	0.1687(2)	0.3373(5)	-0.10941(4)	1.53(2)	0.5	12	12	1.0	2.99(9)
Fe3 (4*f*)$_{Oh}$	0.33333	0.66667	0.19023(7)	1.53(2)	0.16667	4	4	1.0	-3.4(1)
Fe4 (4*f*)$_{Td}$	0.33333	0.66667	0.02706(9)	1.53(2)	0.16667	4	4	1.0	3.8(2)
Fe5 (4*e*)$_{BP}$	0	0	0.2593(2)	1.53(2)	0.08333	4	2	0.5	3.0(2)
O1 (*4e*)	0	0	0.1495(2)	1.14(3)	0.16667	4	4	1.0	
O2 (*4f*)	0.3333	0.6667	-0.0535(2)	1.14(3)	0.16667	4	4	1.0	
O3 (*6h*)	0.1835(9)	0.367(2)	0.25	1.14(3)	0.25	6	6	1.0	
O4 (*12k*)	0.1593(7)	0.319(1)	0.0528(1)	1.14(3)	0.5	12	12	1.0	
O5 (*12k*)	0.5045(8)	0.009(2)	0.1534(1)	1.14(3)	0.5	12	12	1.0	

SSM x=1 ($SrFe_{11}AlO_{19}$)

Refinement profile

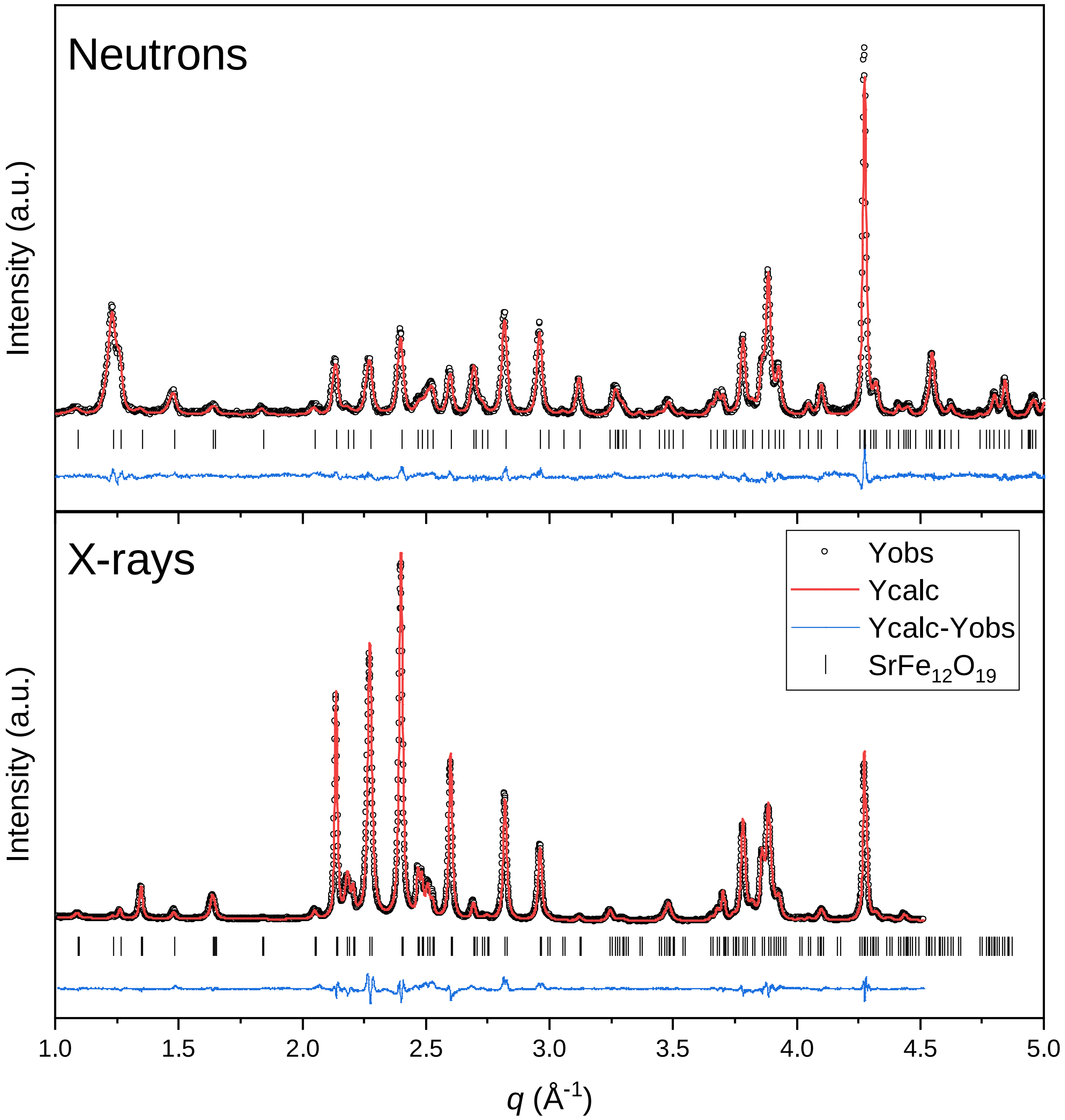

Figure S8

Refined parameters

Phase N. 1: $SrFe_{12-x}Al_xO_{19}$
Space group: P 63/m m c (194); general multiplicity: 24
Phase fraction: 100(1)% Bragg R-factor: 6.66 RF-factor: 4.25 <- Pat#1
Phase fraction: 100.0(5)% Bragg R-factor: 1.81 RF-factor: 1.67 <- Pat#2
Composition: Sr2 Fe23.500(24) Al0.500(24) O38 Theoretical density: 5.077 g/cm3
a = 5.88062(4)Å, b = 5.88062(4)Å, c = 23.0366(3)Å, α = 90.00° β = 90.00° γ = 120.000°
Y= 0.1699(9)
S_z= 2.46(3)

**Atom & site**	**x**	**y**	**z**	$\boldsymbol{B_{iso}}$ **(Å²)**	**Occ.**	**Mult.**	**Compos.**	**Fraction**	$\boldsymbol{R_z\ (\mu_B)}$
Sr (2*d*)	0.3333	0.6667	0.75	2.96(8)	0.08333	2	2	1	
Fe1 $(2a)_{Oh}$	0	0	0	1.62(3)	0.0752(7)	2	1.80(2)	0.902(9)	3.8(3)
Al1 $(2a)_{Oh}$	0	0	0	1.62(3)	0.0082(7)	2	0.20(2)	0.098(9)	
Fe2 $(12k)_{Oh}$	0.1681(3)	0.3362(6)	-0.10925(5)	1.62(3)	0.4873(7)	12	11.70(2)	0.975(1)	2.7(1)
Al2 $(12k)_{Oh}$	0.1681(3)	0.3362(6)	-0.10925(5)	1.62(3)	0.0127(7)	12	0.30(2)	0.025(1)	
Fe3 $(4f)_{Oh}$	0.33333	0.66667	0.1907(1)	1.62(3)	0.16667	4	4	1	-3.5(1)
Al3 $(4f)_{Oh}$	0.33333	0.66667	0.1907(1)	1.62(3)	0	4	0	0	
Fe4 $(4f)_{Td}$	0.33333	0.66667	0.0275(1)	1.62(3)	0.16667	4	4	1	-3.6(2)
Al4 $(4f)_{Td}$	0.33333	0.66667	0.0275(1)	1.62(3)	0	4	0	0	
Fe5 $(4e)_{BP}$	0	0	0.2597(4)	1.62(3)	0.08333	4	2	0.5	2.7(3)
Al5 $(4e)_{BP}$	0	0	0.2597(4)	1.62(3)	0	4	0	0	
O1 (*4e*)	0	0	0.1483(3)	0.99(4)	0.16667	4	4	1	
O2 (*4f*)	0.3333	0.6667	-0.0556(3)	0.99(4)	0.16667	4	4	1	
O3 (*6h*)	0.181(1)	0.361(2)	0.25	0.99(4)	0.25	6	6	1	
O4 (*12k*)	0.1579(9)	0.316(2)	0.0520(1)	0.99(4)	0.5	12	12	1	
O5 (*12k*)	0.5060(9)	0.012(2)	0.1520(1)	0.99(4)	0.5	12	12	1	

SSM x=2 ($SrFe_{10}Al_2O_{19}$)

Refinement profile

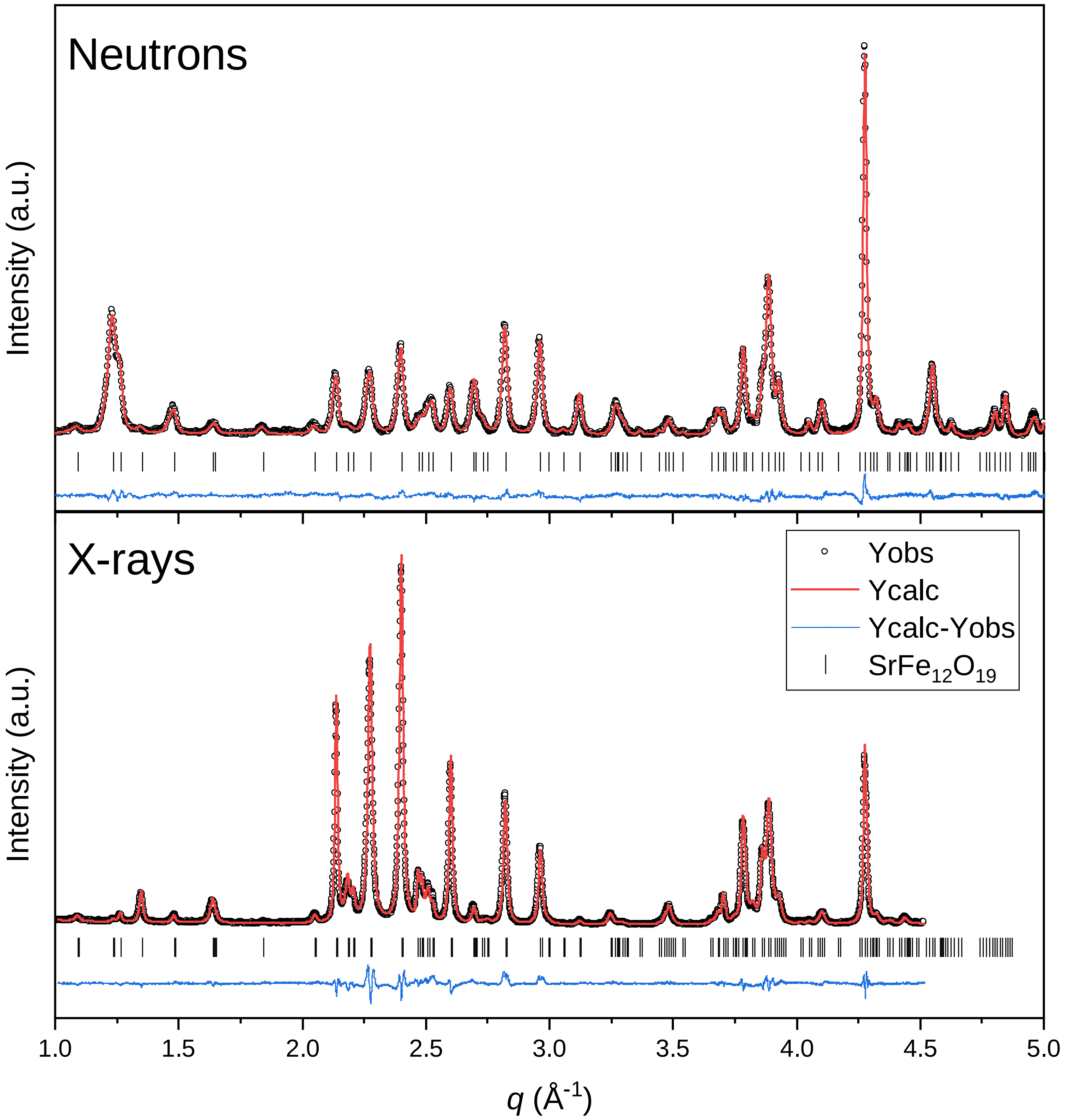

Figure S9

Refined parameters

Phase N. 1: $SrFe_{12-x}Al_xO_{19}$
Space group: P 63/m m c (194); general multiplicity: 24
Phase fraction: 100.0(9)% Bragg R-factor: 5.15 RF-factor: 3.32 <- Pat#1
Phase fraction: 100.0(6)% Bragg R-factor: 1.93 RF-factor: 1.72 <- Pat#2
Composition: Sr2 Fe23.200(26) Al0.800(26) O38 Theoretical density: 5.062 g/cm3
a = 5.87847(5)Å, b = 5.87847(5)Å, c = 23.0268(3)Å, α = 90.00° β = 90.00° γ = 120.000°
Y= 0.178(1)
S_z= 2.52(3)

**Atom & site**	**x**	**y**	**z**	***B*$_{iso}$ (Å^2)**	**Occ.**	**Mult.**	**Compos.**	**Fraction**	***R*$_z$ (μ_B)**
Sr (2*d*)	0.3333	0.6667	0.75	2.39(8)	0.08333	2	2	1	
Fe1 (2*a*)$_{Oh}$	0	0	0	1.14(3)	0.0722(8)	2	1.73(2)	0.866(9)	4.2(2)
Al1 (2*a*)$_{Oh}$	0	0	0	1.14(3)	0.0112(8)	2	0.27(2)	0.134(9)	
Fe2 (12*k*)$_{Oh}$	0.1682(3)	0.3362(7)	-0.10936(5)	1.14(3)	0.4779(8)	12	11.47(2)	0.956(2)	2.93(8)
Al2 (12*k*)$_{Oh}$	0.1682(3)	0.3362(7)	-0.10936(5)	1.14(3)	0.0221(8)	12	0.53(2)	0.044(2)	
Fe3 (4*f*)$_{Oh}$	0.33333	0.66667	0.1906(1)	1.14(3)	0.16667	4	4	1	-3.6(1)
Al3 (4*f*)$_{Oh}$	0.33333	0.66667	0.1906(1)	1.14(3)	0	4	0	0	
Fe4 (4*f*)$_{Td}$	0.33333	0.66667	0.0277(1)	1.14(3)	0.16667	4	4	1	-3.8(1)
Al4 (4*f*)$_{Td}$	0.33333	0.66667	0.0277(1)	1.14(3)	0	4	0	0	
Fe5 (4*e*)$_{BP}$	0	0	0.2600(3)	1.14(3)	0.08333	4	2	0.5	2.6(2)
Al5 (4*e*)$_{BP}$	0	0	0.2600(3)	1.14(3)	0	4	0	0	
O1 (*4e*)	0	0	0.1489(3)	0.68(4)	0.16667	4	4	1	
O2 (*4f*)	0.3333	0.6667	-0.0549(3)	0.68(4)	0.16667	4	4	1	
O3 (*6h*)	0.181(1)	0.361(2)	0.25	0.68(4)	0.25	6	6	1	
O4 (*12k*)	0.1576(9)	0.315(2)	0.0521(1)	0.68(4)	0.5	12	12	1	
O5 (*12k*)	0.5058(10)	0.012(2)	0.1519(1)	0.68(4)	0.5	12	12	1	

SSM x=3 ($SrFe_{19}Al_3O_{19}$)

Refinement profile

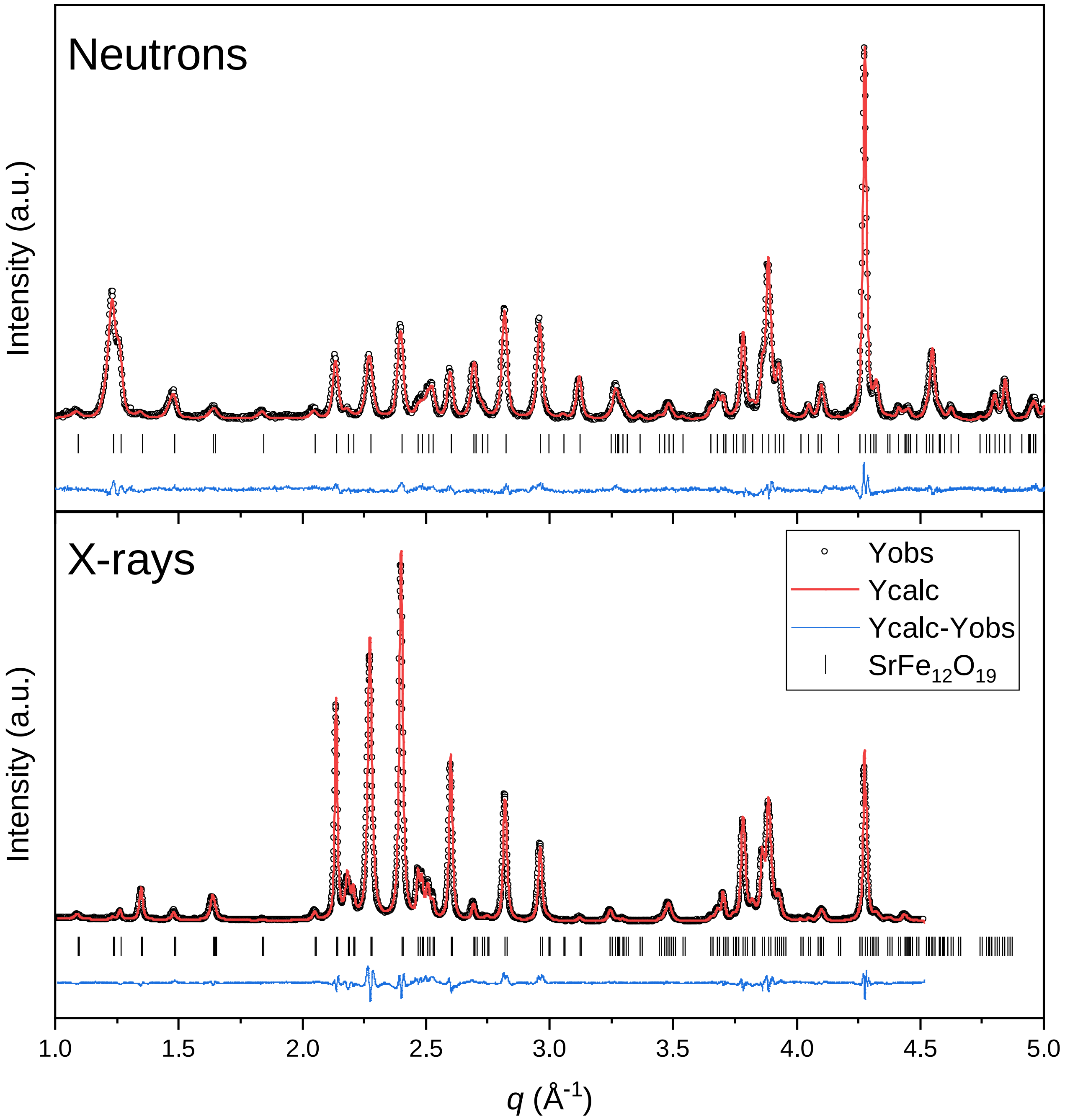

Figure S10

Refined parameters

Phase N. 1: $SrFe_{12-x}Al_xO_{19}$
Space group: P 63/m m c (194); general multiplicity: 24
Phase fraction: 100(1)% Bragg R-factor: 6.17 RF-factor: 3.86 <- Pat#1
Phase fraction: 100.0(5)% Bragg R-factor: 1.94 RF-factor: 1.73 <- Pat#2
Composition: Sr2 Fe23.401(24) Al0.599(24) O38 Theoretical density: 5.07 g/cm3
a = 5.88076(5)Å, b = 5.88076(5)Å, c = 23.0351(3)Å, α = 90.00° β = 90.00° γ = 120.000°
Y= 0.177(1)
S_z= 2.27(3)

**Atom & site**	**x**	**y**	**z**	**B_{iso} (Å^2)**	**Occ.**	**Mult.**	**Compos.**	**Fraction**	**R_z (μ_B)**
Sr (2*d*)	0.3333	0.6667	0.75	2.69(8)	0.08333	2	2	1	
Fe1 (2*a*)$_{Oh}$	0	0	0	1.38(3)	0.0742(7)	2	1.78(2)	0.890(9)	4.1(3)
Al1 (2*a*)$_{Oh}$	0	0	0	1.38(3)	0.0092(7)	2	0.22(2)	0.110(9)	
Fe2 (12*k*)$_{Oh}$	0.1684(3)	0.3367(6)	-0.10923(5)	1.38(3)	0.4842(7)	12	11.62(2)	0.968(1)	2.97(9)
Al2 (12*k*)$_{Oh}$	0.1684(3)	0.3367(6)	-0.10923(5)	1.38(3)	0.0158(7)	12	0.38(2)	0.032(1)	
Fe3 (4*f*)$_{Oh}$	0.33333	0.66667	0.19071(9)	1.38(3)	0.16667	4	4	1	-3.6(1)
Al3 (4*f*)$_{Oh}$	0.33333	0.66667	0.19071(9)	1.38(3)	0	4	0	0	
Fe4 (4*f*)$_{Td}$	0.33333	0.66667	0.0275(1)	1.38(3)	0.16667	4	4	1	-3.7(2)
Al4 (4*f*)$_{Td}$	0.33333	0.66667	0.0275(1)	1.38(3)	0	4	0	0	
Fe5 (4*e*)$_{BP}$	0	0	0.2600(3)	1.38(3)	0.08333	4	2	0.5	2.5(2)
Al5 (4*e*)$_{BP}$	0	0	0.2600(3)	1.38(3)	0	4	0	0	
O1 (*4e*)	0	0	0.1492(3)	0.91(4)	0.16667	4	4	1	
O2 (*4f*)	0.3333	0.6667	-0.0549(3)	0.91(4)	0.16667	4	4	1	
O3 (*6h*)	0.181(1)	0.362(2)	0.25	0.91(4)	0.25	6	6	1	
O4 (*12k*)	0.1576(9)	0.315(2)	0.0523(1)	0.91(4)	0.5	12	12	1	
O5 (*12k*)	0.5063(9)	0.013(2)	0.1522(1)	0.91(4)	0.5	12	12	1	

Site Occupation Fraction of Al cations in the SSM samples

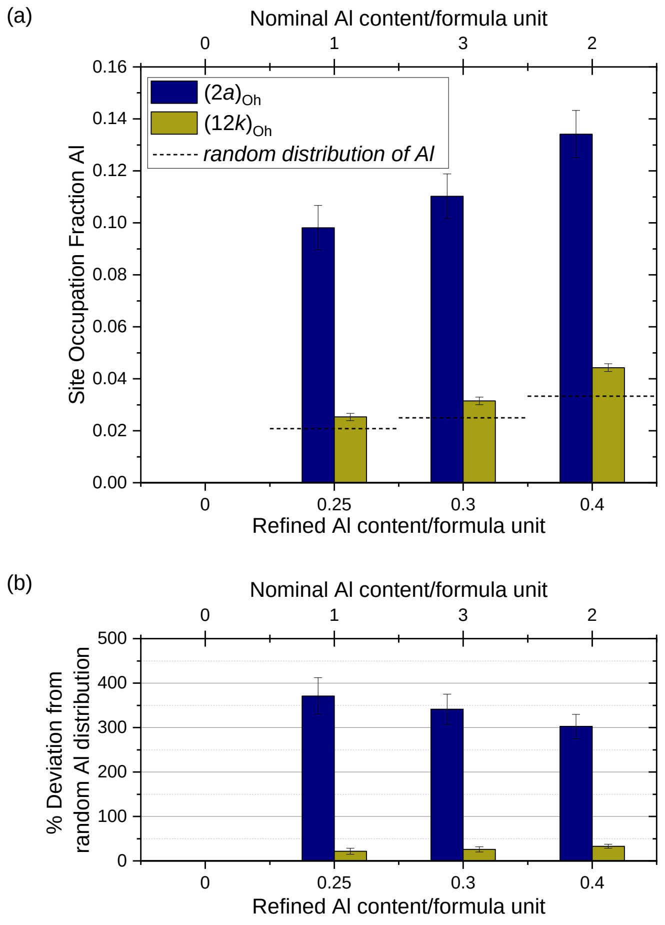

Figure S11: a) Refined site occupation fraction of Al cations in each of the 2 octahedral 2*a* and 12*k* crystallographic sites for the SSM-synthesized samples. b) Percentage deviation from random distribution of Al cations in the 2 occupied sites.

Combined Rietveld refinements of PXRD and NPD data of SG samples

SG x=0 ($SrFe_{12}O_{19}$)

Refinement profile

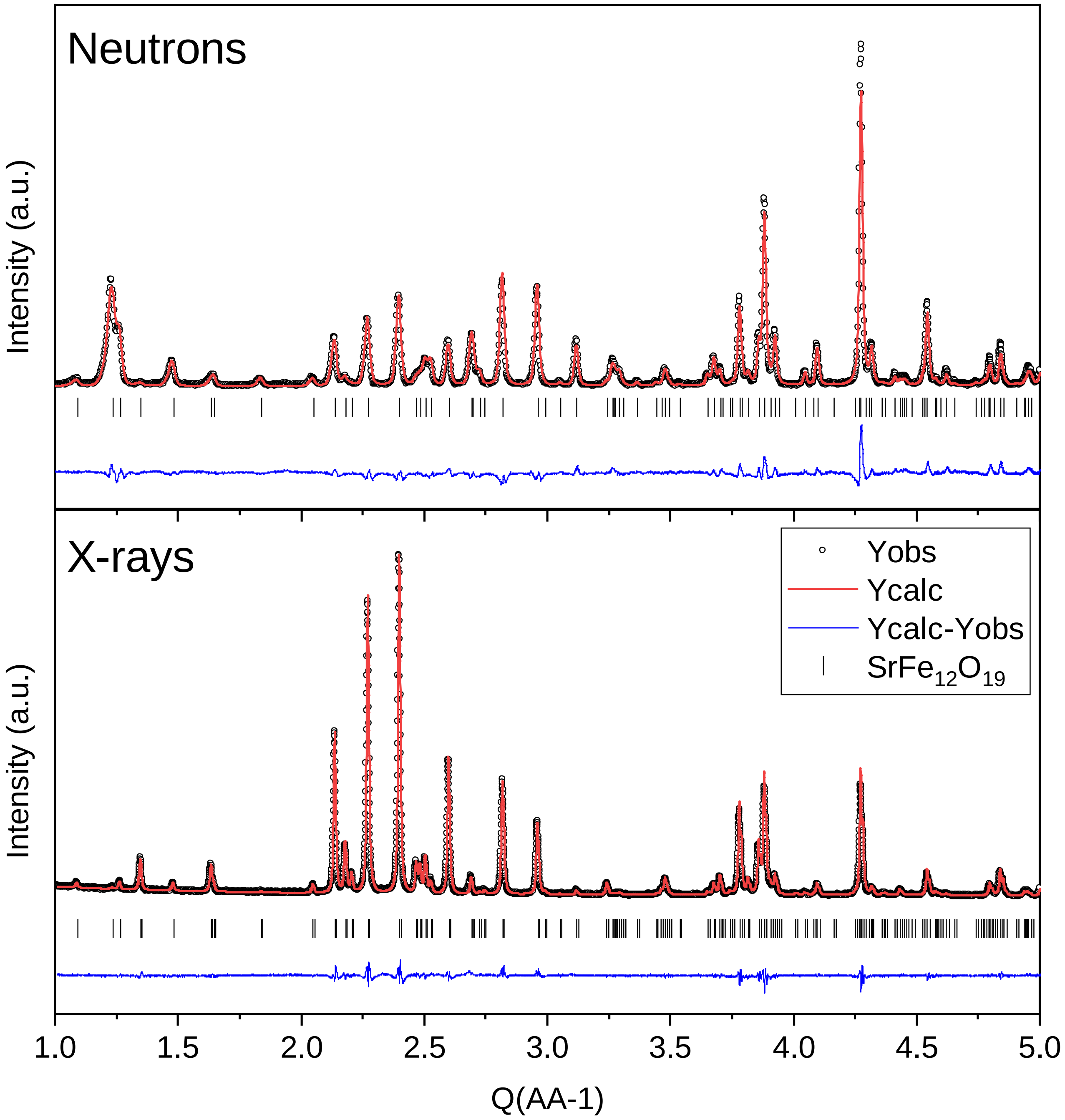

Figure S12

Refined parameters

Phase N. 1: $SrFe_{12}O_{19}$
Space group: P 63/m m c (194); general multiplicity: 24
Phase fraction: 100.0(9)% Bragg R-factor: 7.22 RF-factor: 4.72 <- Pat#1
Phase fraction: 100.0(4)% Bragg R-factor: 5.01 RF-factor: 6.79 <- Pat#2
Composition: Sr2 Fe24 O38 Theoretical density: 5.103 g/cm3
a = 5.88187(3)Å, b = 5.88187(3)Å, c = 23.0654(2)Å, α = 90.00° β = 90.00° γ = 120.000°
Y= 0.1210(9)
S_z= 0.24(2)

**Atom & site**	**x**	**y**	**z**	B_{iso} **(Å^2)**	**Occ.**	**Mult.**	**Compos.**	**Fraction**	R_z (μ_B)
Sr (2*d*)	0.3333	0.6667	0.75	1.77(7)	0.08333	2	2	1.0	
Fe1 (2*a*)$_{Oh}$	0	0	0	1.60(3)	0.08333	2	2	1.0	3.6(2)
Fe2 (12*k*)$_{Oh}$	0.1685(4)	0.3369(7)	-0.10916(6)	1.60(3)	0.5	12	12	1.0	3.00(8)
Fe3 (4*f*)$_{Oh}$	0.33333	0.66667	0.1906(1)	1.60(3)	0.16667	4	4	1.0	-3.38(9)
Fe4 (4*f*)$_{Td}$	0.33333	0.66667	0.0269(1)	1.60(3)	0.16667	4	4	1.0	-3.8(1)
Fe5 (4*e*)$_{BP}$	0	0	0.2571(4)	1.60(3)	0.08333	4	2	0.5	3.8(2)
O1 (*4e*)	0	0	0.1498(3)	1.13(4)	0.16667	4	4	1.0	
O2 (*4f*)	0.3333	0.6667	-0.0548(3)	1.13(4)	0.16667	4	4	1.0	
O3 (*6h*)	0.184(1)	0.367(3)	0.25	1.13(4)	0.25	6	6	1.0	
O4 (*12k*)	0.1583(9)	0.317(2)	0.0526(2)	1.13(4)	0.5	12	12	1.0	
O5 (*12k*)	0.506(1)	0.012(2)	0.1514(1)	1.13(4)	0.5	12	12	1.0	

SG x=1 ($SrFe_{11}AlO_{19}$)

Refinement profile

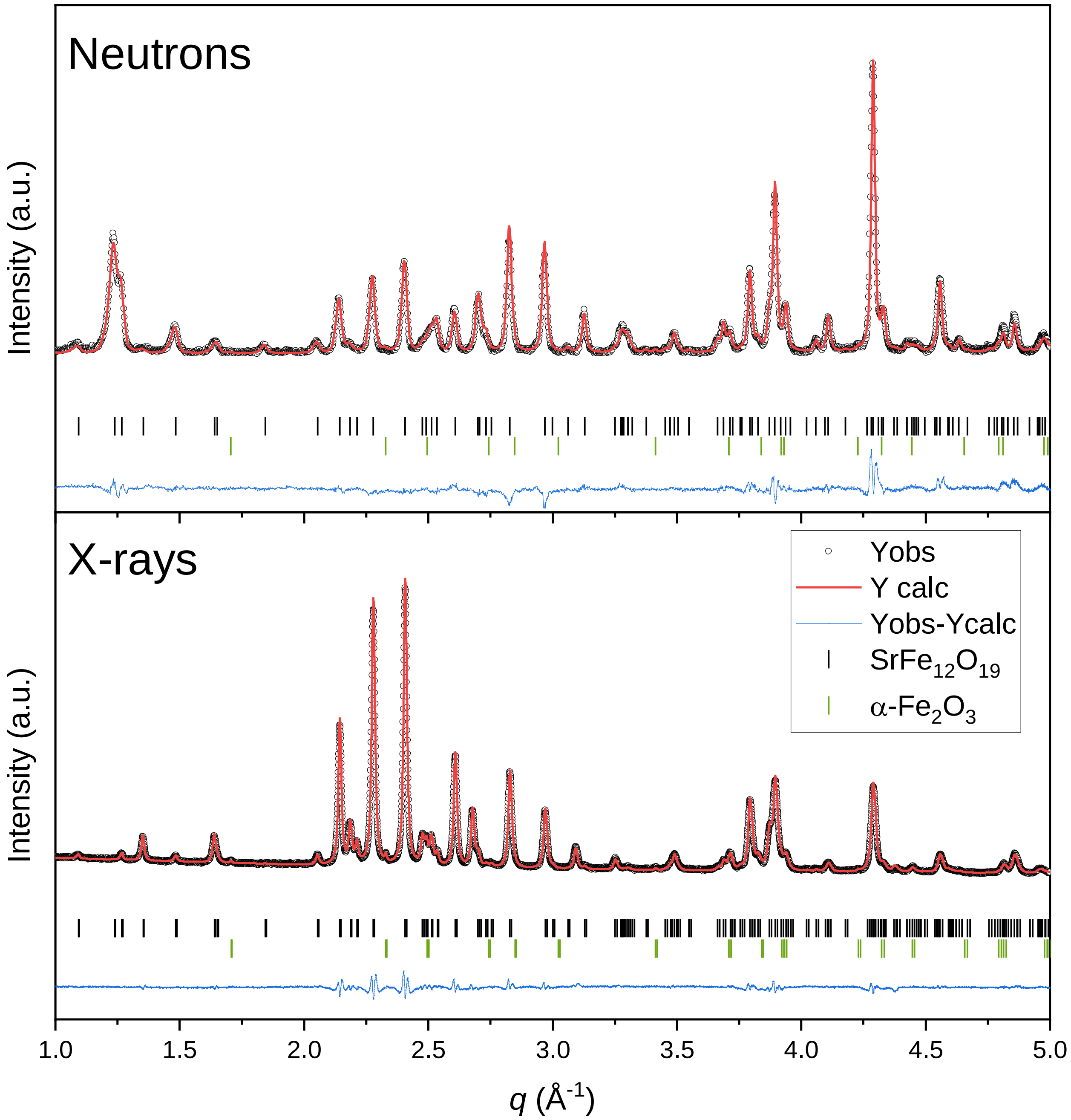

Figure S13

Refined parameters

Phase N. 1: $SrFe_{12-x}Al_xO_{19}$
Space group: P 63/m m c (194); general multiplicity: 24
Phase fraction: 99(1)% Bragg R-factor: 8.62 RF-factor: 5.15 <- Pat#1
Phase fraction: 99.3(6)% Bragg R-factor: 3.89 RF-factor: 4.19 <- Pat#2
Composition: Sr2 Fe22.001(64) Al1.999(64) O38 Theoretical density: 5.017 g/cm3
a = 5.86027(5)Å, b = 5.86027(5)Å, c = 22.9901(3)Å, α = 90.00° β = 90.00° γ = 120.000°
Y= 0.147(3)
S_z= 0.30(3)
X= 0.582(6)

**Atom & site**	**x**	**y**	**z**	B_{iso} **(Å²)**	**Occ.**	**Mult.**	**Compos.**	**Fraction**	R_z (μ_B)
Sr (2*d*)	0.3333	0.6667	0.75	2.01(8)	0.08333	2	2	1	
Fe1 (2*a*)$_{Oh}$	0	0	0	1.93(3)	0.0603(10)	2	1.45(2)	0.72(1)	5.0(4)
Al1 (2*a*)$_{Oh}$	0	0	0	1.93(3)	0.0230(10)	2	0.55(2)	0.28(1)	
Fe2 (12*k*)$_{Oh}$	0.1683(4)	0.3367(8)	-0.10888(6)	1.93(3)	0.452(1)	12	10.85(3)	0.904(2)	3.0(1)
Al2 (12*k*)$_{Oh}$	0.1683(4)	0.3367(8)	-0.10888(6)	1.93(3)	0.048(1)	12	1.15(3)	0.096(2)	
Fe3 (4*f*)$_{Oh}$	0.33333	0.66667	0.1906(1)	1.93(3)	0.161(1)	4	3.88(3)	0.969(8)	-3.9(1)
Al3 (4*f*)$_{Oh}$	0.33333	0.66667	0.1906(1)	1.93(3)	0.005(1)	4	0.12(3)	0.031(8)	
Fe4 (4*f*)$_{Td}$	0.33333	0.66667	0.0270(1)	1.93(3)	0.165(1)	4	3.95(3)	0.987(8)	-3.5(2)
Al4 (4*f*)$_{Td}$	0.33333	0.66667	0.0270(1)	1.93(3)	0.002(1)	4	0.05(3)	0.013(8)	
Fe5 (4*e*)$_{BP}$	0	0	0.2565(7)	1.93(3)	0.078(1)	4	1.88(3)	0.470(7)	2.6(3)
Al5 (4*e*)$_{BP}$	0	0	0.2565(7)	1.93(3)	0.005(1)	4	0.12(3)	0.030(7)	
O1 (*4e*)	0	0	0.1498(3)	0.98(4)	0.16667	4	4	1	
O2 (*4f*)	0.33333	0.66667	-0.0557(3)	0.98(4)	0.16667	4	4	1	
O3 (*6h*)	0.184(1)	0.368(3)	0.25	0.98(4)	0.25	6	6	1	
O4 (*12k*)	0.159(1)	0.317(2)	0.0513(1)	0.98(4)	0.5	12	12	1	
O5 (*12k*)	0.505(1)	0.011(2)	0.1511(2)	0.98(4)	0.5	12	12	1	

Phase N. 2: α-Fe_2O_3
Space group: R -3 c (167); general multiplicity: 36
Phase fraction: 1.3(2)% Bragg R-factor: 14.10 RF-factor: 7.54 <- Pat#1
Phase fraction: 0.71(6)% Bragg R-factor: 10.50 RF-factor: 6.50 <- Pat#2
Composition: Fe12 O18 Theoretical density: 5.274 g/cm3
a = 5.0376(5)Å, b = 5.0376(5)Å, c = 13.727(2)Å, α = 90.00° β = 90.00° γ = 120.000°
Y=0.09(2)

**Atom & site**	**x**	**y**	**z**	B_{iso} **(Å²)**	**Occ.**	**Mult.**	**Compos.**	**Fraction**
Fe (12*c*)	0	0	0.35522	1.93(3)	0.33333	12	12	1.0
O (18*e*)	0.69395	0	0.25	0.98(4)	0.5	18	18	1.0

SG x=2 ($SrFe_{10}Al_2O_{19}$)

Refinement profile

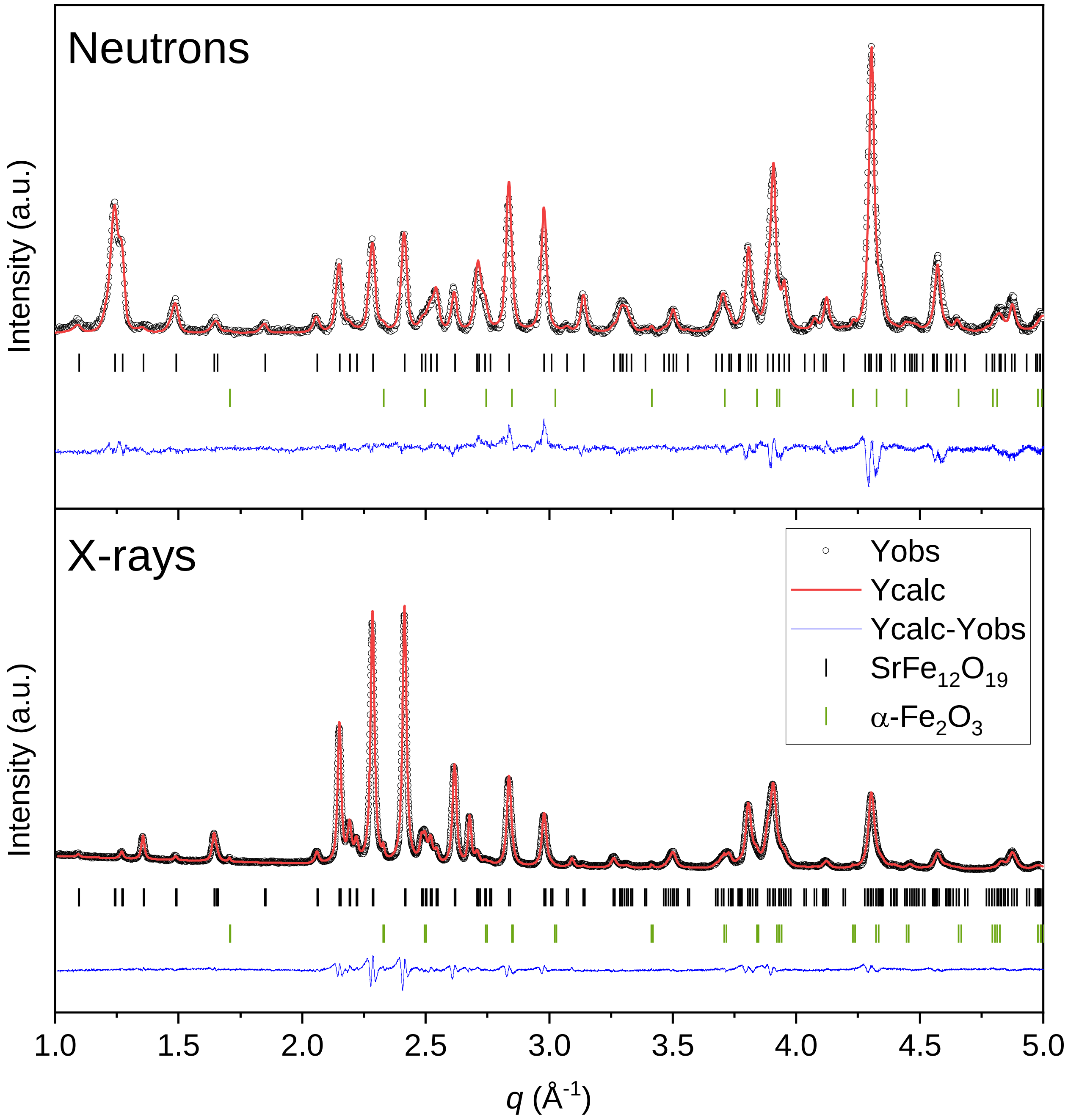

Figure S14

Refined parameters

Phase N. 1: $SrFe_{12-x}Al_xO_{19}$
Space group: P 63/m m c (194); general multiplicity: 24
Phase fraction: 98(1)% Bragg R-factor: 7.55 RF-factor: 4.10 <- Pat#1
Phase fraction: 99(4)% Bragg R-factor: 4.71 RF-factor: 4.14 <- Pat#2
Composition: Sr2 Fe20.001(72) Al3.999(72) O38 Theoretical density: 4.92 g/cm3
a = 5.84179(8)Å, b = 5.84179(8)Å, c = 22.9317(4)Å, α = 90.00° β = 90.00° γ = 120.000°
Y= 0.100(4)
S_z= 0.38(4)
X= 0.33(1)

**Atom & site**	**x**	**y**	**z**	**B_{iso} (Å^2)**	**Occ.**	**Mult.**	**Compos.**	**Fraction**	**R_z (μ_B)**
Sr (2*d*)	0.3333	0.6667	0.75	1.83(9)	0.08333	2	2	1	
Fe1 (2*a*)$_{Oh}$	0	0	0	1.81(4)	0.048(1)	2	1.15(3)	0.57(1)	5.3(5)
Al1 (2*a*)$_{Oh}$	0	0	0	1.81(4)	0.035(1)	2	0.85(3)	0.43(1)	
Fe2 (12*k*)$_{Oh}$	0.1683(5)	0.337(1)	-0.10844(7)	1.81(4)	0.397(1)	12	9.54(3)	0.795(3)	3.0(2)
Al2 (12*k*)$_{Oh}$	0.1683(5)	0.337(1)	-0.10844(7)	1.81(4)	0.103(1)	12	2.46(3)	0.205(3)	
Fe3 (4*f*)$_{Oh}$	0.33333	0.66667	0.1895(2)	1.81(4)	0.154(1)	4	3.69(3)	0.923(8)	-3.9(2)
Al3 (4*f*)$_{Oh}$	0.33333	0.66667	0.1895(2)	1.81(4)	0.013(1)	4	0.31(3)	0.077(8)	
Fe4 (4*f*)$_{Td}$	0.33333	0.66667	0.0268(2)	1.81(4)	0.162(2)	4	3.89(4)	0.973(10)	-3.7(2)
Al4 (4*f*)$_{Td}$	0.33333	0.66667	0.0268(2)	1.81(4)	0.004(2)	4	0.11(4)	0.027(10)	
Fe5 (4*e*)$_{BP}$	0	0	0.249(6)	1.81(4)	0.072(1)	4	1.73(3)	0.432(8)	2.3(3)
Al5 (4*e*)$_{BP}$	0	0	0.249(6)	1.81(4)	0.011(1)	4	0.27(3)	0.068(8)	
O1 (*4e*)	0	0	0.1500(4)	0.67(5)	0.16667	4	4	1	
O2 (*4f*)	0.33333	0.66667	-0.0552(4)	0.67(5)	0.16667	4	4	1	
O3 (*6h*)	0.184(2)	0.368(3)	0.25	0.67(5)	0.25	6	6	1	
O4 (*12k*)	0.158(1)	0.316(2)	0.0518(2)	0.67(5)	0.5	12	12	1	
O5 (*12k*)	0.506(1)	0.012(3)	0.1506(2)	0.67(5)	0.5	12	12	1	

Phase N. 2: α-Fe_2O_3
Space group: R -3 c (167); general multiplicity: 36
Phase fraction: 1.8(2)% Bragg R-factor: 13.10 RF-factor: 6.97 <- Pat#1
Phase fraction: 0.64(6)% Bragg R-factor: 8.76 RF-factor: 5.80 <- Pat#2
Composition: Fe12 O18 Theoretical density: 5.275 g/cm3
a = 5.034618Å, b = 5.034618Å, c = 13.741706Å, α = 90.00° β = 90.00° γ = 120.000°
Y= 0.10(1)

**Atom & site**	**x**	**y**	**z**	**B_{iso} (Å^2)**	**Occ.**	**Mult.**	**Compos.**	**Fraction**
Fe (12*c*)	0	0	0.35522	1.80(4)	0.33333	12	12	1.0
O (18*e*)	0.69395	0	0.25	0.79(5)	0.5	18	18	1.0

SG x=3 ($SrFe_9Al_3O_{19}$)

Refinement profile

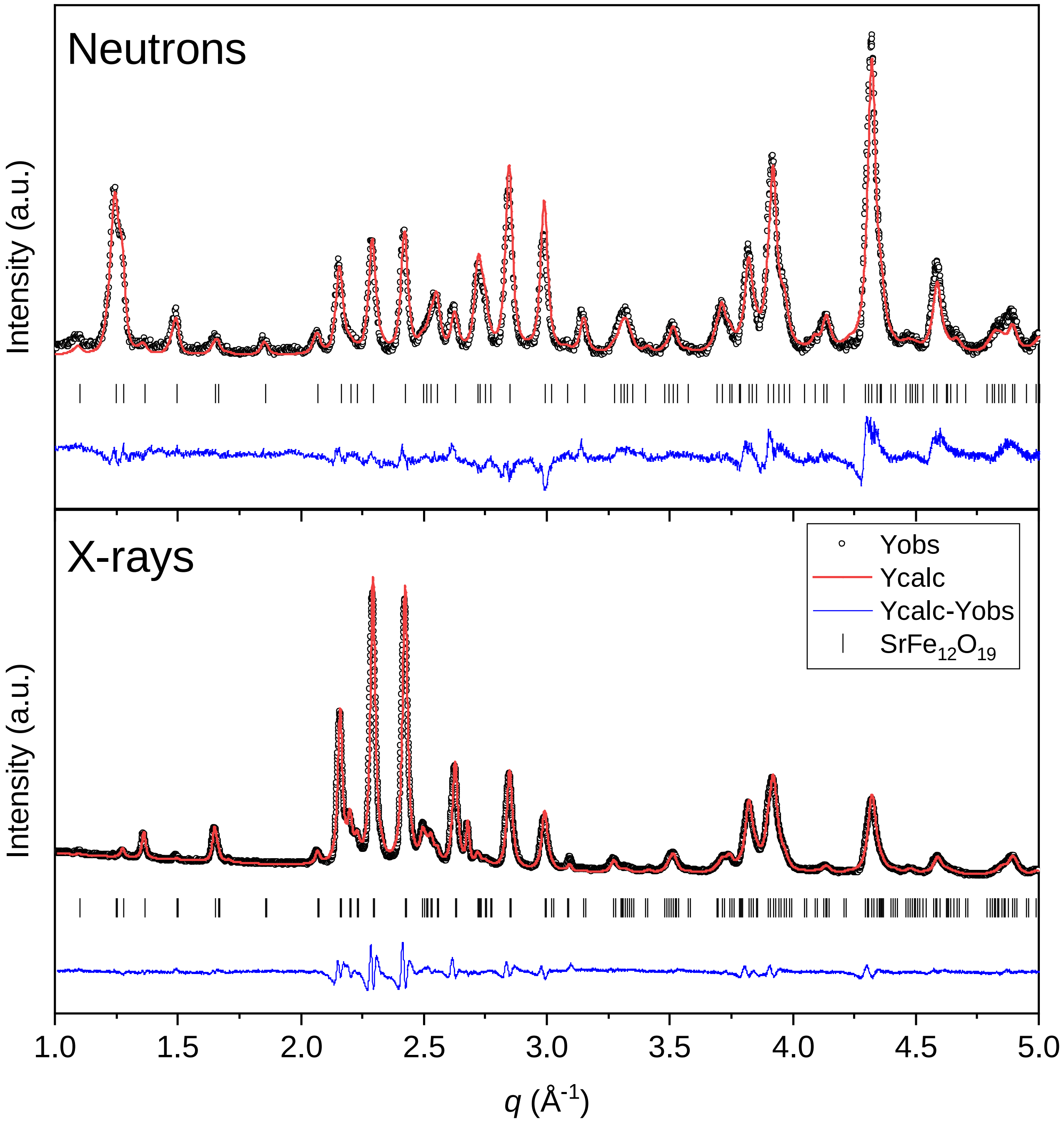

Figure 15

Refined parameters

Phase N. 1: $SrFe_{12-x}Al_xO_{19}$
Space group: P 63/m m c (194); general multiplicity: 24
Phase fraction: 98(2)% Bragg R-factor: 8.35 RF-factor: 4.71 <- Pat#1
Phase fraction: 99.3(3)% Bragg R-factor: 3.67 RF-factor: 3.62 <- Pat#2
Composition: Sr2 Fe18.003(94) Al5.997(94) O38 Theoretical density: 4.832 g/cm3
a = 5.8190(1)Å, b = 5.8190(1)Å, c = 22.8574(6)Å, α = 90.00° β = 90.00° γ = 120.000°
Y= 0.103(7)
S_z= 0.13(7)
X= 0.75(2)

**Atom & site**	**x**	**y**	**z**	B_{iso} **(Å^2)**	**Occ.**	**Mult.**	**Compos.**	**Fraction**	R_z (μ_B)
Sr (2*d*)	0.3333	0.6667	0.75	1.2(1)	0.08333	2	2	1	
Fe1 (2*a*)$_{Oh}$	0	0	0	1.81(5)	0.045(2)	2	1.07(4)	0.54(2)	5.4(6)
Al1 (2*a*)$_{Oh}$	0	0	0	1.81(5)	0.039(2)	2	0.93(4)	0.46(2)	
Fe2 (12*k*)$_{Oh}$	0.1687(8)	0.337(2)	-0.1082(1)	1.81(5)	0.339(2)	12	8.13(4)	0.678(4)	3.0(2)
Al2 (12*k*)$_{Oh}$	0.1687(8)	0.337(2)	-0.1082(1)	1.81(5)	0.161(2)	12	3.87(4)	0.322(4)	
Fe3 (4*f*)$_{Oh}$	0.33333	0.66667	0.1892(2)	1.81(5)	0.137(2)	4	3.29(5)	0.82(1)	-3.8(2)
Al3 (4*f*)$_{Oh}$	0.33333	0.66667	0.1892(2)	1.81(5)	0.030(2)	4	0.71(5)	0.18(1)	
Fe4 (4*f*)$_{Td}$	0.33333	0.66667	0.0270(2)	1.81(5)	0.165(2)	4	3.95(5)	0.99(1)	-3.6(3)
Al4 (4*f*)$_{Td}$	0.33333	0.66667	0.0270(2)	1.81(5)	0.002(2)	4	0.05(5)	0.01(1)	
Fe5 (4*e*)$_{BP}$	0	0	0.25	1.81(5)	0.065(1)	2	1.56(3)	0.78(2)	1.9(4)
Al5 (4*e*)$_{BP}$	0	0	0.25	1.81(5)	0.018(1)	2	0.44(3)	0.22(2)	
O1 (*4e*)	0	0	0.1480(5)	0.58(7)	0.16667	4	4	1	
O2 (*4f*)	0.33333	0.66667	-0.0544(5)	0.58(7)	0.16667	4	4	1	
O3 (*6h*)	0.185(2)	0.370(4)	0.25	0.58(7)	0.25	6	6	1	
O4 (*12k*)	0.159(2)	0.318(3)	0.0521(2)	0.58(7)	0.5	12	12	1	
O5 (*12k*)	0.507(2)	0.013(4)	0.1501(2)	0.58(7)	0.5	12	12	1	

Phase N. 2: α-Fe_2O_3
Space group: R -3 c (167); general multiplicity: 36
Phase fraction: 1.93(2)% Bragg R-factor: 13.50 RF-factor: 6.65 <- Pat#1
Phase fraction: 0.66% Bragg R-factor: 10.40 RF-factor: 5.94 <- Pat#2
Composition: Fe12 O18 Theoretical density: 5.275 g/cm3
a = 5.034618Å, b = 5.034618Å, c = 13.741706Å, α = 90.00° β = 90.00° γ = 120.000°
Y = 0.46(4)

**Atom & site**	**x**	**y**	**z**	B_{iso} **(Å^2)**	**Occ.**	**Mult.**	**Compos.**	**Fraction**
Fe (12*c*)	0	0	0.35522	2.01557	0.33333	12	12	1.0
O (18*e*)	0.69395	0	0.25	0.25094	0.5	18	18	1.0

Refined magnetic moments as function of nominal Al content

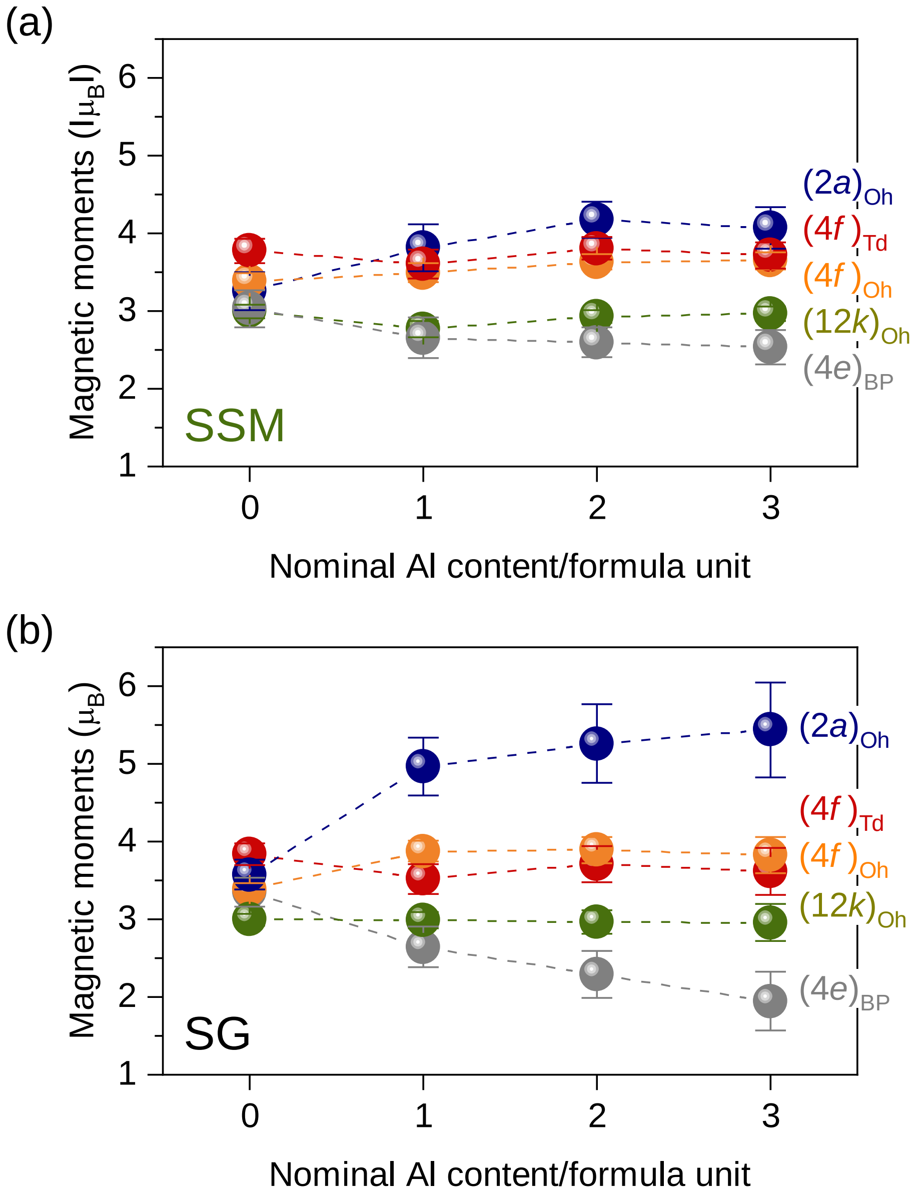

Figure S16: Refined magnetic moments of Fe^{3+} on the five different crystallographic Fe^{3+} sites as function of increasing nominal aluminum content, for samples synthesized by (a) SSM and (b) SG methods.

Refined and measured magnetization as function of nominal Al content

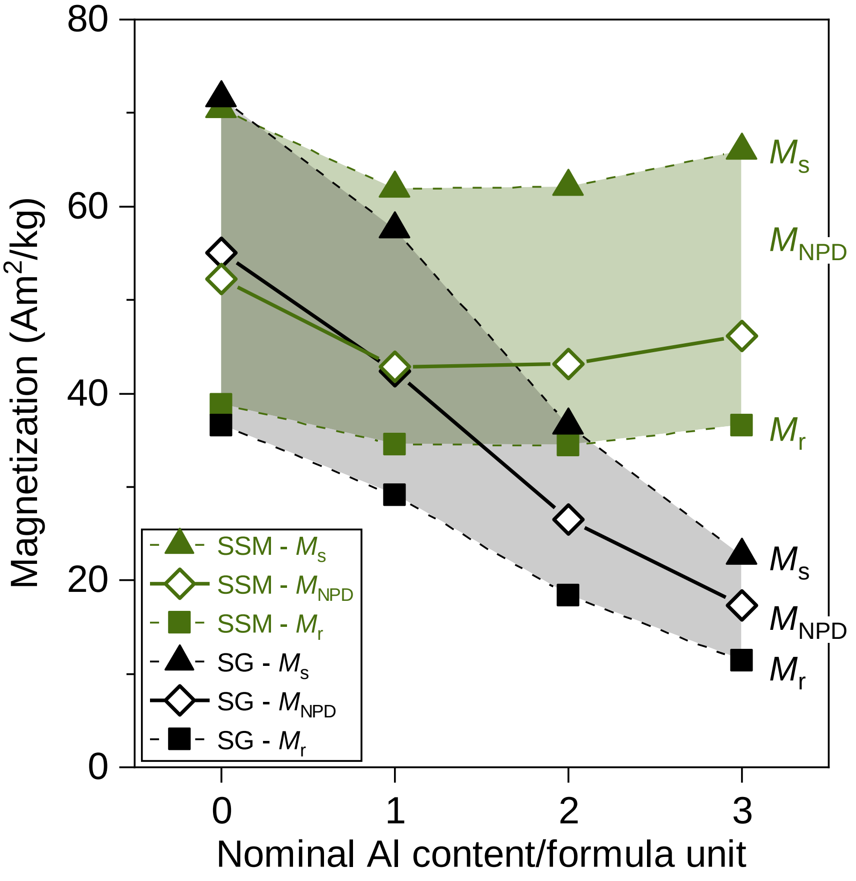

Figure S17: Measured saturation magnetization (M_s), remanent magnetization (M_r) and calculated magnetization from the refined magnetic moments of the NPD data (M_{NPD}) for both, the SG and SSM series, as function of nominal Al substitution. Error bars within the size of the symbols.
